# Plakoglobin phosphorylation at serine 665 is capable of stabilizing cadherin-mediated adhesion in keratinocytes

**DOI:** 10.1172/jci.insight.190359

**Published:** 2026-02-09

**Authors:** Franziska Vielmuth, Anna M. Sigmund, Desalegn T. Egu, Matthias Hiermaier, Letyfee S. Steinert, Sina Moztarzadeh, Mariia Klimkina, Margarethe E.C. Schikora, Paulina M. Rion, Thomas Schmitt, Katharina Meier, Kamran Ghoreschi, Anja K.E. Horn, Mariya Y. Radeva, Daniela Kugelmann, Jens Waschke

**Affiliations:** 1 Chair of Vegetative Anatomy, Institute of Anatomy, Faculty of Medicine, LMU Munich, Munich, Germany.; 2 Department of Dermatology, Venereology and Allergology, Charité-Universitätsmedizin Berlin, Berlin, Germany.

**Keywords:** Autoimmunity, Cell biology, Autoimmune diseases, Cell migration/adhesion

## Abstract

In pemphigus, autoantibodies against the desmosomal cadherins desmoglein (DSG) DSG1 and DSG3 cause intraepidermal blistering. Recently, we found that increasing cAMP with the phosphodiesterase-4 inhibitor apremilast stabilizes keratinocyte cohesion in pemphigus. This effect is paralleled by phosphorylation of the desmosomal plaque protein plakoglobin (PG) at serine 665 (S665). Here, we investigated the relevance of PG phosphorylation at S665 for stabilization of keratinocyte cohesion and further characterized the underlying mechanisms. Ultrastructural analysis of a recently established PG-S665 phospho-deficient mouse model (PG-S665A) showed diminished keratin insertion. Accordingly, the protective effect of apremilast against pemphigus autoantibody-induced skin blistering was diminished, and apremilast failed to restore alterations of the keratin cytoskeleton in PG-S665A mice. Keratinocytes derived from PG-S665A mice revealed a disorganized keratin cytoskeleton and reduced single-molecule binding strength of DSG3. In line with this, in ex vivo human skin, increased cAMP augmented keratin insertion into desmosomal plaques. Additionally, PG phosphorylated at S665 colocalized with desmoplakin and keratin filaments anchoring to desmosomes and increased cAMP-accelerated assembly of desmosomes. Taken together, phosphorylation of PG at S665 was crucial for protective effects of apremilast in pemphigus and for maintenance of DSG3 binding and keratin filament anchorage to desmosomes.

## Abstract 2

Phosphorylation of Pg at S665 is crucial for protective effects of apremilast in pemphigus and for maintenance of Dsg3 binding and keratin filament anchorage to desmosomes.

## Graphical abstract

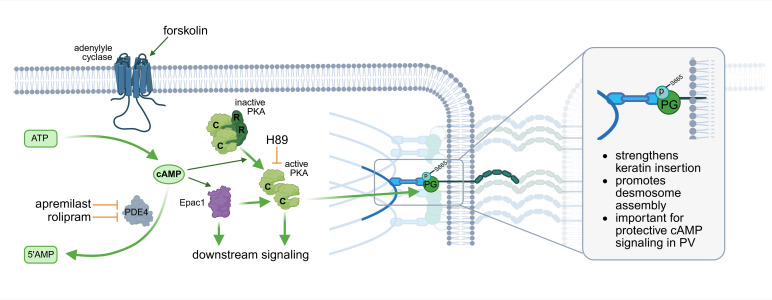

## Introduction

In the epidermis, desmosomes maintain epidermal stability in the face of mechanical stress by providing strong intercellular adhesion (1). On a molecular level, they consist of desmosomal cadherins, which maintain adhesive strength with their extracellular domains. Intracellularly, desmosomal cadherins are linked via several plaque proteins, such as desmoplakin (DP) and plakoglobin (PG), to the intermediate filament cytoskeleton, which in the epidermis are represented by keratins (2, 3). PG interacts with both desmosomal and classical cadherins and thus can be found in desmosomes as well as in adherens junctions (4, 5). Moreover, PG was reported to critically contribute to assembly of the desmosomal plaque, and it was found to be involved in anchorage of the desmosome to the keratin cytoskeleton (6–8). In addition, a cadherin-independent pool of PG fulfills several cellular functions and is involved in intracellular signaling. Previously, we reported that extranuclear PG regulates cell adhesion via p38MAPK-dependent regulation of the keratin cytoskeleton (9).

Pemphigus is a life-threatening bullous autoimmune disease in which autoantibodies against the desmosomal cadherins, desmoglein (DSG) DSG1 and DSG3, cause flaccid blistering of the epidermis and the mucous membranes of the oral cavity (10, 11). Dysregulation of a plethora of signaling pathways, such as p38MAPK, ERK, PKC, and SRC, contribute to loss of intercellular adhesion in pemphigus (12). In contrast, pemphigus vulgaris–IgGs (PV-IgGs) increase intracellular cAMP level, which seems to reflect an insufficient cellular rescue mechanism that can be pharmacologically augmented (13). Thus, cAMP increase stabilizes cadherin-mediated keratinocyte cohesion even in the presence of pemphigus autoantibodies. Although previously used compounds such as the adenylate cyclase activator forskolin and the β-adrenergic receptor agonist isoprenaline drastically elevate cAMP levels, they cannot be applied in patients due to expectable severe side effects. In contrast, the PDE4 inhibitor apremilast is clinically approved for skin diseases such as psoriasis and is effective in protecting keratinocytes from loss of intercellular adhesion caused by pemphigus autoantibodies (14, 15). In addition, apremilast has already been used in case studies to treat patients with pemphigus successfully (16–18). In a previous study, apremilast stabilizes keratinocyte cohesion in vitro, ex vivo in human skin, and in vivo in mice by preventing keratin retraction. This mechanism involves phosphorylation of PG at serine 665 (S665) (15), a phosphorylation site that also stabilizes cardiomyocyte cohesion in a PKA-dependent manner (19).

In clinical practice, first-line therapy of pemphigus focuses on suppression of the immune system including corticosteroids, immunosuppressive drugs, and rituximab (20, 21). However, therapeutic strategies directly strengthening desmosomal adhesion in the presence of autoantibodies would be strongly beneficial, especially in the acute stage of disease and may lead to a therapeutic regimen with less side effects. Thus, a profound understanding of the mechanisms of PG phosphorylation at S665 involved in protective cAMP signaling and regulation of desmosomal adhesion is of high clinical relevance. To this end, we here investigate the mechanisms by which PG phosphorylation at S665 is capable of stabilizing cadherin-mediated adhesion in keratinocytes using mice phospho-deficient for PG at S665 (PG-S665A), as well as using keratinocytes derived from these mice.

## Results

### PG phosphorylation at S665 is crucial for protective cAMP signaling in pemphigus.

**Figure 1:**
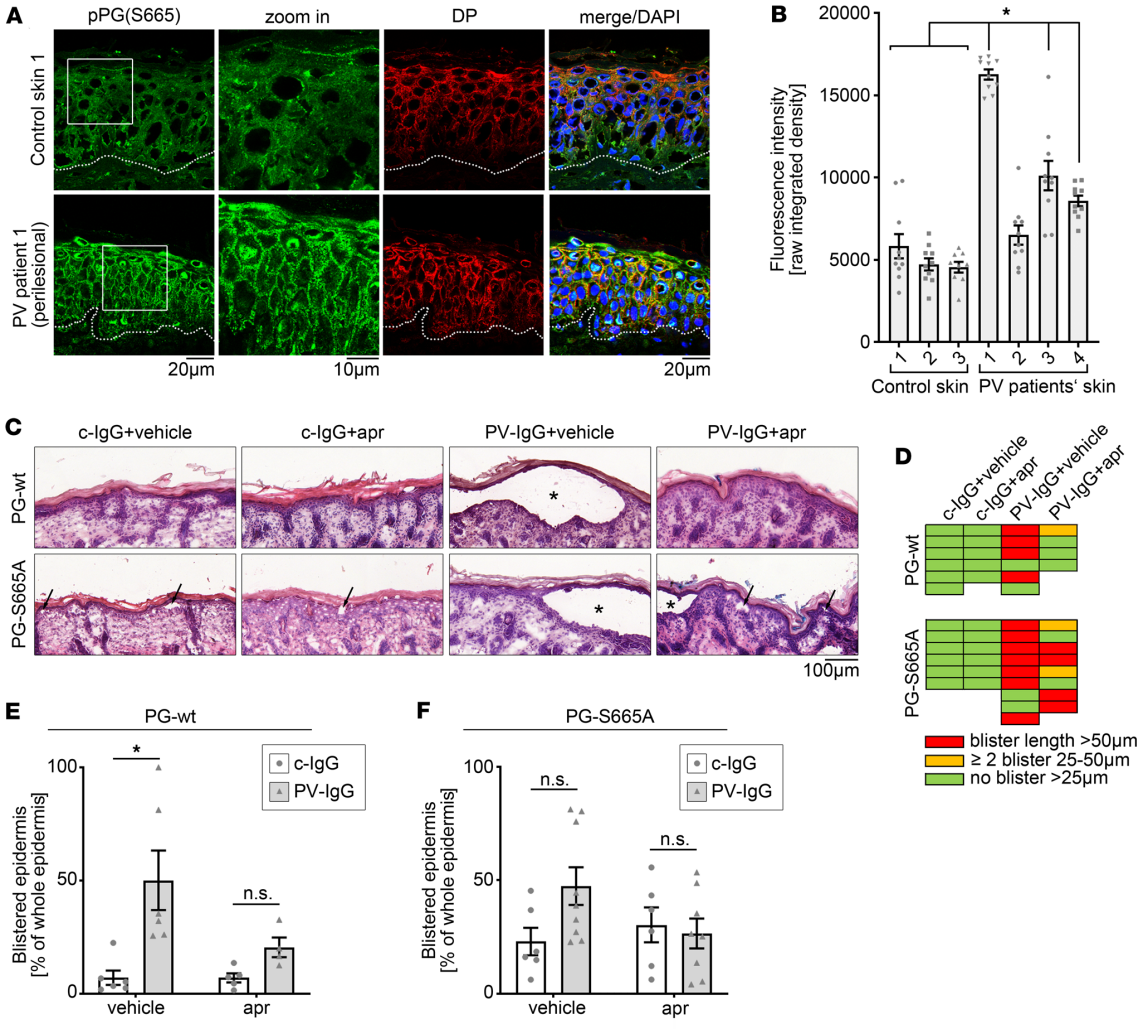
Phosphorylation of PG at S665 protects keratinocytes from PV-IgG–induced loss of intercellular adhesion. (**A**) Immunostaining of pPG(S665) and DP in control skin 1 (pemphigus-negative) and perilesional skin of PV patient 1. White dotted lines represent basement membrane of epidermis. PG phosphorylation at S665 is enhanced along cell borders in perilesional skin of PV patient. DP costaining was used as desmosome marker. Representative of *n* = 3–4. Scale bars: 10 μm; 20 μm. (**B**) Quantification of immunostaining in **A** confirms significant increase of PG at S665 along cell borders in 3 of 4 PV patients’ skin samples compared with every control sample; 10 cell borders/skin sample. (**C**) H&E staining of neonatal PV mouse model in PG-WT and PG-S665A mice. PV-IgG induced blistering in PG-WT and PG-S665A mice, which was abrogated by apr in PG-WT mice only. Representative of *n* = 5–9. Asterisk represents big blister larger than 50 μm. Black arrows show areas with micro-blisters smaller than 25 μm. Scale bar: 100 μm. (**D**) Analysis of occurrence of big blisters with split length > 25 μm (blister length > 50 μm [red], 25–50 μm [orange], no blister > 25 μm [green]). Whereas no big blisters occurred in PG-WT and PG-S665A mice under control IgG conditions, PV-IgG led to blister formation in PG-WT and PG-S665A mice, which was efficiently blocked by apremilast in PG-WT but only slightly improved in PG-S665A. (**E** and **F**) Quantification of blistered epidermis (including small blisters <25 μm and micro-blisters) in PG-WT (**E**) and PG-S665A (**F**) mice revealed significant increased blistering in PV-IgG–treated mice in PG-WT and only a trend toward higher blister formation in PG-S665A, where baseline adhesion was reduced. Apr prevented blistering in PG-WT but only slightly improved PV-IgG–induced blistering in PG-S665A. *n* = 4–9, data shown as mean ± SEM. One-way ANOVA with Dunnett’s post hoc test (**B**); 2-way ANOVA with Tukey’s post hoc test (**E** and **F**); *P < 0.05; apr, apremilast; DP, desmoplakin; PG, plakoglobin.

An increase of cAMP levels in keratinocytes was observed after incubation with IgG from patients with PV (PV-IgG) in keratinocytes and represents an insufficient rescue mechanism (22). Pharmacological augmentation, for example, by the PDE4 inhibitor apremilast, effectively prevents PV-IgG–induced loss of intercellular adhesion and involves phosphorylation of PG at S665 (15). Thus, we investigated whether phosphorylation of PG at S665 is altered in the skin of patients with PV. To do so, biopsies of control skin and perilesional skin from patients with pemphigus were stained for pPG (S665) and DP. In control skin samples, phosphorylation of PG at S665 was only present to a minor extent and barely visible along keratinocyte cell borders (Figure 1A). In contrast, phosphorylation was significantly elevated along cell borders in 3 out of 4 analyzed skin samples from patients with pemphigus (Figure 1, A and B). Taken together, the data revealed that PG was more strongly phosphorylated at S665 along junctions in perilesional epidermis of patients with pemphigus, indicating an involvement of PG phosphorylation at S665 in a cAMP-mediated rescue mechanism.

Thus, we here aimed to investigate the role of PG phosphorylation at S665 in pemphigus. We first applied a neonatal pemphigus mouse model in PG-WT mice and compared the effects of PV-IgG incubation and apremilast treatment with PG-S665A mice, in which a serine at position 665 was replaced by alanine and thus the mice were phospho-deficient at this position (15). These mice have an altered expression pattern of desmosomal proteins in the skin, and keratinocytes derived from these mice have a drastically impaired intercellular adhesion (15). Nevertheless, neither thickness nor layering of the epidermis was altered drastically in PG-S665A mice (15). PG-WT and PG-S665A mice were injected with apremilast (1 μM) or vehicle (DMSO; 1:10,000 in PBS) 2 hours prior to injection with control IgG fraction from healthy donors (c-IgG) or PV-IgG, respectively. Subsequently, mice were euthanized after 10 hours to harvest the skin for further analysis. H&E staining of the skin showed an intact skin after c-IgG injection with and without apremilast in the skin of PG-WT animals, whereas micro-blisters were observed in PG-S665A mice under both conditions (Figure 1C). In WT mice, PV-IgG injection induced loss of intercellular adhesion, causing typical epidermal blistering in 4 out of 6 mice, which was prevented by coinjection with apremilast in 3 out of 4 mice (Figure 1, C and D). In addition, skin slices were analyzed with respect to all blistered areas including small blisters/micro-blisters. In PG-WT animals, PV-IgG drastically increased blistering in the epidermis, which was significantly blocked by apremilast treatment (Figure 1E), confirming the protective effect of apremilast against PV-IgG–induced loss of intercellular adhesion. In PG-S665A mice, baseline adhesion was impaired, as shown before (15). Therefore, PV-IgG treatment in PG-S665A mice did not significantly increase overall blistered area (Figure 1F), although it effectively induced gross blistering in 7 out of 9 animals (Figure 1, C and D). Cotreatment with apremilast only slightly reduced formation of big blisters, which was only rescued in 2 out of 8 animals (Figure 1D) and did not significantly prevent overall blistering (Figure 1F). Taken together, these data show that PG phosphorylation at S665 is crucial for the protective effect of apremilast in pemphigus.

**Figure 2:**
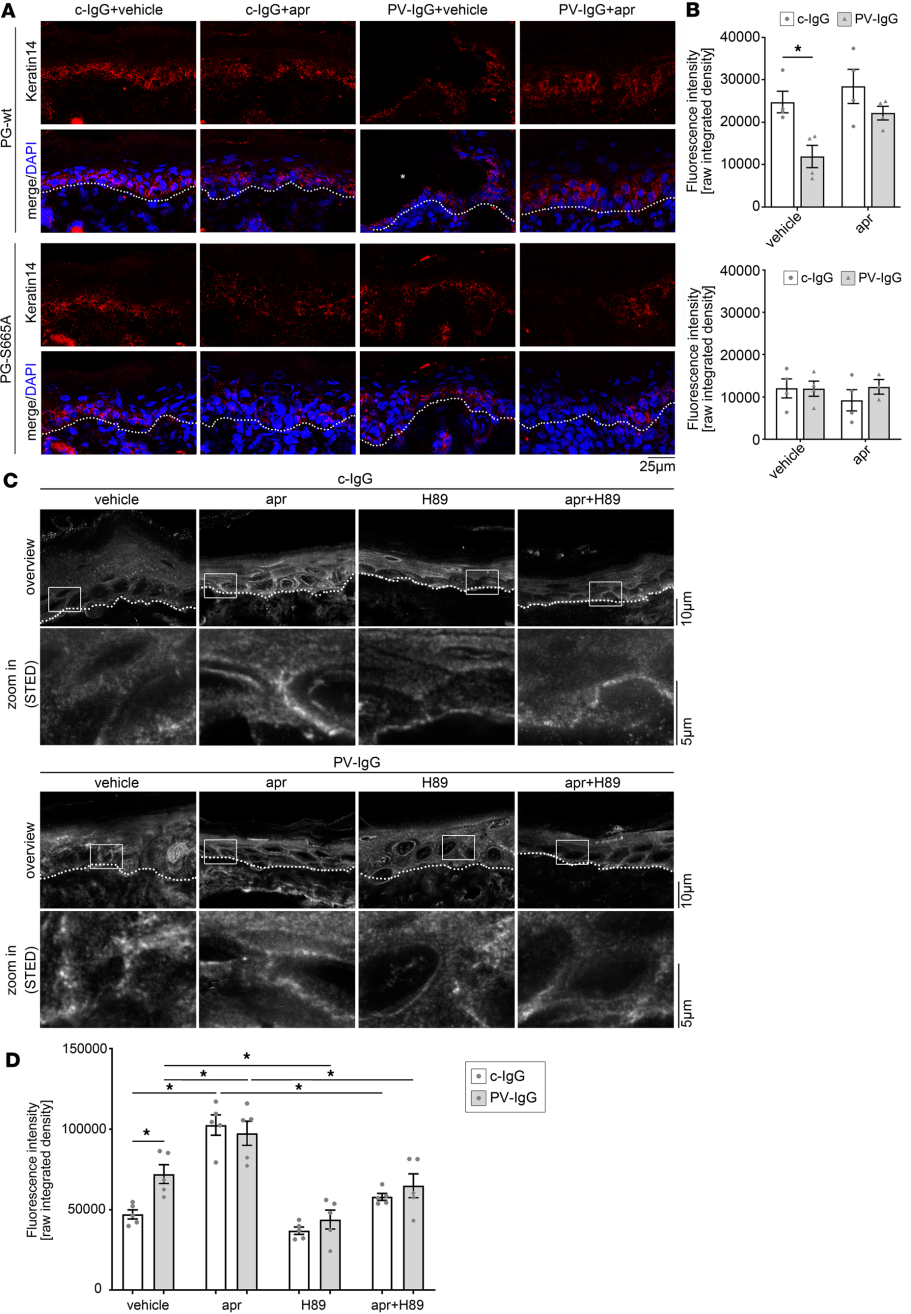
Apremilast abolishes PV-IgG–induced keratin alterations. (**A**) Immunostaining of keratin 14 in PG-WT and PG-S665A mice from neonatal PV mouse model. DAPI was used to identify overall tissue morphology by nuclei staining. Basement membrane is marked with a punctuated white line and blister with an asterisk. Keratin 14, which was expressed in the basal and suprabasal epidermal layers, was compromised upon PV-IgG injection. Apr ameliorated PV-IgG–induced keratin alteration. Representative of *n* = 4. Scale bar: 25 μm. (**B**) Quantification of **A**. PV-IgG significantly reduced fluorescence intensity in PG-WT mice, which was absent by coincubation with apr. In contrast, baseline fluorescence levels were diminished in PG-S665A mice, and neither PV-IgG nor apr induced significant changes in keratin 14 fluorescence intensity. *n* = 4. Data shown as mean ± SEM. Two-way ANOVA with Tukey’s post hoc test, *P < 0.05. (**C** and **D**) STED imaging of pPG(S665) in human ex vivo skin model. Apr and the PKA inhibitor H89 were preincubated for 1 hour. Subsequently, c-IgG or PV-IgG were injected and incubated for another 1 hour. PV-IgGs and apr increased phosphorylation of PG at S665 along cell borders. Apr had an additive effect on PV-IgG–induced increase. H89 ameliorated PV-IgG–induced and apr-induced phosphorylation of PG at S665. Representative of *n* = 5. Scale bars: 5 μm; 10 μm. (**D**) Quantification of STED imaging in **C**. *n* = 5, 2-way ANOVA with Tukey’s post hoc test; *P < 0.05; apr, apremilast; c-IgG, IgG fraction of healthy control; PG, plakoglobin.

Apremilast abolished PV-IgG–induced alteration of the keratin cytoskeleton in vitro in keratinocytes as well as in ex vivo human skin (15). Thus, we wondered whether apremilast also prevents keratin alteration in the murine pemphigus model. Cryosections of the respective condition were stained for keratin 14. In PG-WT mice, keratin 14 was expressed in basal and suprabasal layers of the epidermis as described before (1). Mice injected with PV-IgG revealed blister formation accompanied by reduction of overall keratin 14 staining, which was rescued by apremilast (Figure 2, A and B). In contrast, keratin 14 was drastically reduced in the epidermis of PG-S665A mice injected with c-IgG (Figure 2, A and B). PV-IgG did not further reduce keratin 14 immunostaining, and importantly, apremilast had no effect on the keratin cytoskeleton organization in these mice (Figure 2, A and B).

Next, we used an ex vivo human skin model to investigate whether PV-IgG directly leads to PG phosphorylation at S665 and whether cAMP increase by apremilast has an additional effect. We observed that PV-IgG significantly increased PG phosphorylation at S665, which is in accordance with the cAMP increase observed in previous studies (22). Importantly, apremilast had an additional effect on PG phosphorylation at S665 in both ex vivo samples incubated with c-IgG or PV-IgG (Figure 2, C and D). To gain further insights into the mechanisms underlying PG phosphorylation, we used the PKA inhibitor H89. H89 blocked PG phosphorylation at S665 induced by both PV-IgGs and apremilast, showing that PG phosphorylation at S665 is PKA dependent and thus most likely caused by cAMP signaling (Figure 2, C and D).

### cAMP signaling affects keratin insertion into desmosomes in keratinocytes.

**Figure 3:**
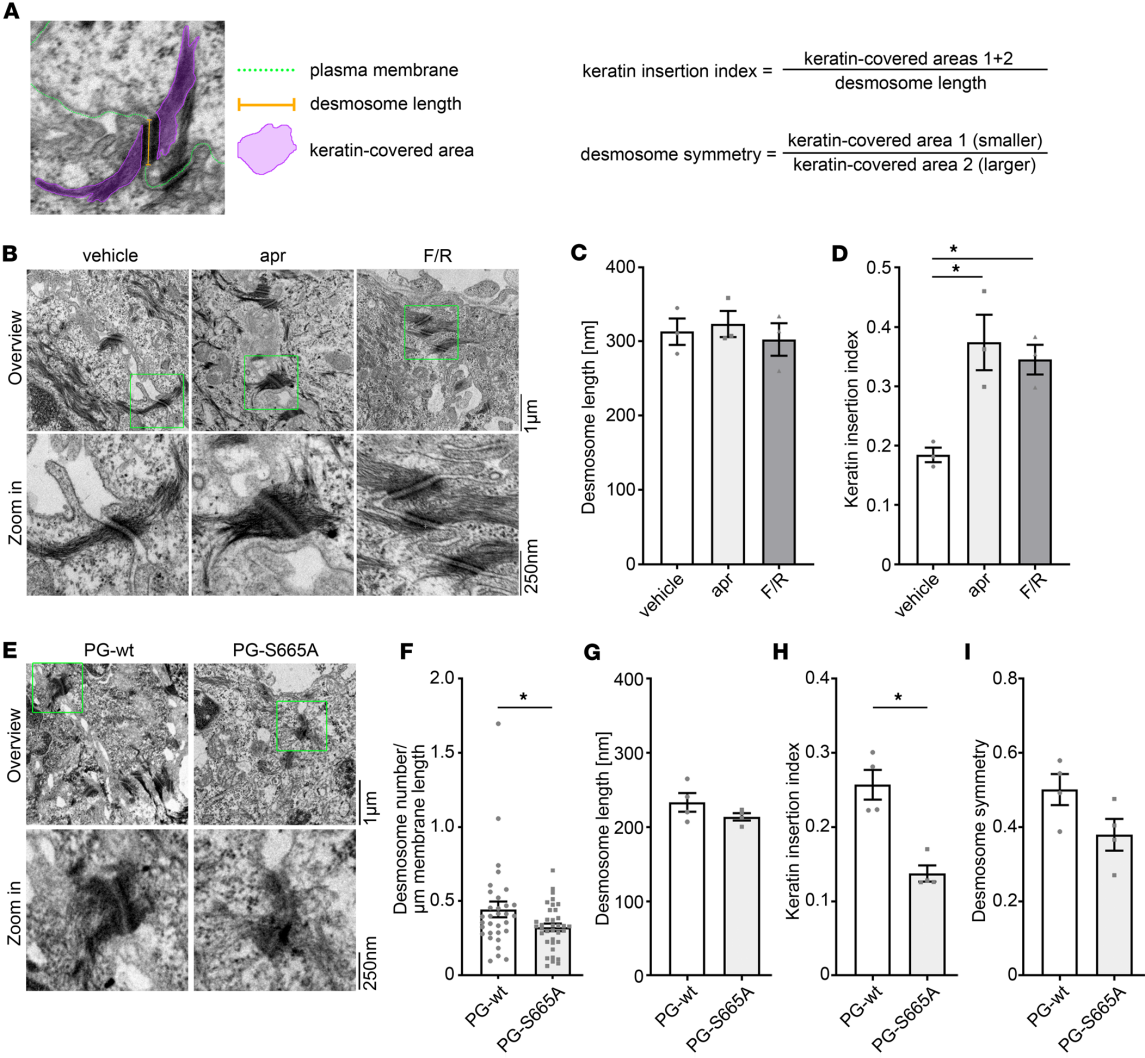
Keratin insertion into desmosomes is dependent on cAMP signaling. (**A**) Schematic of analysis of desmosome ultrastructure in electron micrographs; 30–50 desmosomes were analyzed. (**B**) Electron micrograph from human ex vivo skin model showing desmosomes connecting keratinocytes in the basal/suprabasal layer of the epidermis. Human skin was injected for 1 hour with vehicle, forskolin/rolipram (F/R), or apremilast (apr). cAMP increase led to augmented keratin insertion into desmosomes. Zoom-ins are marked with green rectangles in overview micrographs. Representative of *n* = 3. Scale bars: 1 μm; 250 nm. (**C** and **D**) Quantification of desmosome length (**C**) and keratin insertion (**D**) from human ex vivo skin model in **B** reveals that F/R did not change desmosome length but confirms that keratin insertion is significantly increased in keratin insertion index. *n* = 3. (**E**) Electron micrograph from murine skin of PG-WT and PG-S665A animals reveals altered keratin insertion in PG-S665A mice epidermis. Zoom-ins are marked with green rectangles in overview micrographs. Representative of *n* = 4. Scale bars: 1 μm; 250 nm. (**F**) Quantification of desmosome number from **E** with 10 cell borders/*n*. (**G**) Quantification of **E** reveals no difference in desmosome length between PG-WT and PG-S665A. (**H**) Keratin insertion index calculated from means of 30–40 desmosomes/*n* from **E** showed significant impaired keratin insertion in PG-S665A mice epidermis. (**I**) Quantification of desmosome symmetry in PG-WT and PG-S665A mice epidermis showed a trend toward impaired symmetry in PG-S665A mice epidermis; 30–40 desmosomes/*n*. (**G**–**I**) *n* = 4; data shown as mean ± SEM. One-way ANOVA with Tukey’s post hoc test (**C** and **D**), unpaired 2-tailed *t* test (**G** and **I**), Mann-Whitney *U* test (**F** and **H**). *P < 0.05.

Keratin anchorage is crucial for proper adhesive function of desmosomes in keratinocytes (23, 24). This is also reflected in loss of intercellular adhesion in pemphigus, where alterations of the keratin cytoskeleton, ultrastructurally visible by uncoupling of keratins from the desmosomal plaque, represent a morphological hallmark in pemphigus (25–27). Recently, we observed that apremilast abolished PV-IgG–induced keratin filament alterations in vitro and in vivo (Figure 2, A and B) (15). Thus, we questioned whether an increase of cAMP in keratinocytes affects ultrastructure of desmosomes and keratin anchorage. To do so, desmosomes were analyzed in electron micrographs with respect to desmosome number as well as desmosome length, keratin insertion, and symmetry, as shown in Figure 3A. Human ex vivo skin samples were treated for 1 hour with apremilast or a combination of the adenylyl cyclase activator forskolin and the PDE4 inhibitor rolipram (F/R) and processed for electron microscopy. Neither apremilast nor F/R changed the length of the desmosomes (Figure 3, B and C). In contrast, insertion of keratins was significantly increased upon cAMP elevation by apremilast and F/R (Figure 3, B and D), showing that cAMP signaling regulates proper keratin insertion into desmosomes. To analyze whether these alterations are dependent on PG phosphorylation at S665, we next compared the ultrastructure of desmosomes in PG-WT and PG-S665A mice epidermis. In these mice, the overall structure of the epidermis was not altered in H&E staining (Figure 1C), the number of desmosomes was slightly decreased in PG-phospho-deficient mice, and desmosome length remained unaltered (Figure 3, E–G). Next, we checked for keratin insertion, which was drastically impaired in PG-S665A mice (Figure 3, E and H), indicating that pPG(S665) is important for keratin insertion into desmosomes. Additionally, desmosomes of PG-S665A mice revealed a trend toward less symmetry (Figure 3, E and I), which can be regarded as a measure for desmosomes with proper adhesive function.

### PG phosphorylation at S665 promotes recruitment of keratins to assembling desmosomes.

Previous ultrastructural data showed that pPG(S665) is crucial for proper insertion of keratins into desmosomes (Figure 3). Thus, we next asked whether PG phosphorylation at S665 is involved in desmosome turnover. First, we performed stimulated emission depletion (STED) experiments in murine keratinocytes derived from PG-WT or PG-S665A mice with mature desmosomes (48 hours in 1.2 mM Ca^2+^ medium) and characterized the colocalization pattern of pPG and other desmosomal components (Supplemental Figure 1, A–E; supplemental material available online with this article; https://doi.org/10.1172/jci.insight.190359DS1). As expected, the desmosomal plaque proteins DP and PG colocalized along the cell borders in both PG-WT and PG-S665A keratinocytes, although overall staining of DP was reduced in PG-S665A keratinocytes (Supplemental Figure 1, A, B, and D). pPG(S665), which was absent in PG-S665A keratinocytes, colocalized to DP in mature desmosomes (Supplemental Figure 1B). Next, we stained the plaque proteins together with keratin 14. PG and DP were located along keratin 14 filaments inserting to the cell-cell contact area in PG-WT keratinocytes (Supplemental Figure 1, C and D). Here, pPG(S665) was also present along keratin 14 filaments inserting to cell-cell contact areas (Supplemental Figure 1E). In contrast, keratin 14 organization was severely disturbed in PG-S665A keratinocytes, and keratin 14 filaments inserting to cell border areas were rare (Supplemental Figure 1, C–E). Taken together, these data show that pPG(S665) is located to the cell membrane where it is colocalized with desmosomal markers such as DP. Next, we wondered whether phosphorylation of PG at S665 changes the interaction of PG with other junctional proteins. First, we used immunoprecipitation of PG even though levels of both PG and DP were reduced in PG-S665A keratinocytes. Nevertheless, the relative amount of DP interacting with PG was not significantly different between PG-WT and PG-S665A keratinocytes (Supplemental Figure 2, A and B). Similarly, colocalization of PG with DSG3 and DSC3 remained unaltered, as revealed by STED microscopy (Supplemental Figure 2C).

**Figure 4:**
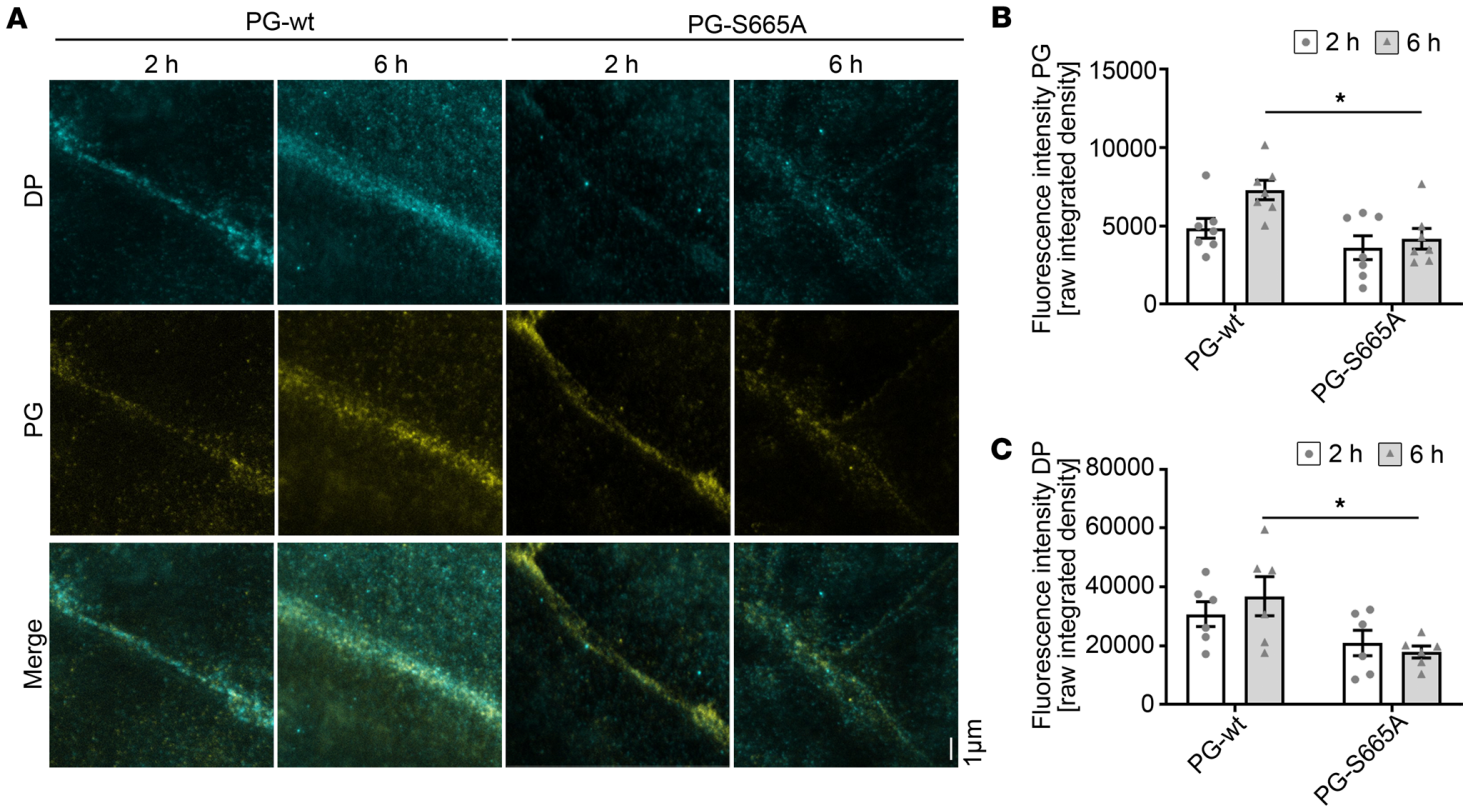
PG phosphorylation at S665 is important for the assembly of desmosomes. (**A**) Costaining of PG and DP in PG-WT and PG-S665A murine keratinocytes after switch to high Ca^2+^ medium for 2 hours and 6 hours. Initial recruitment of PG is not altered after 2 hours but staining is reduced after 6 hours. In accordance, DP staining is reduced at 6 hours of Ca^2+^-induced desmosome assembly. Representative of *n* = 7. Scale bar: 1 μm. (**B** and **C**) Quantification of fluorescence intensity from **A** for PG (**B**) and DP (**C**) along cell borders; *n* = 7, mean of 10 cell borders/*n*. Data shown as mean ± SEM. Two-way-ANOVA with Tukey’s post hoc test (**B** and **C**). *P < 0.05.DP, desmoplakin; PG, plakoglobin.

Next, we asked whether PG phosphorylation at S665 has an impact on the turnover of desmosomes and thus performed assembly experiments. To do so, confluent monolayers of PG-WT and PG-S665A keratinocytes were switched to high Ca^2+^ (1.2 mM) and cultured for 2 or 6 hours before colocalization of PG-DP and PG-CK14 was analyzed. The ability of apremilast to increase cAMP levels during desmosome assembly was checked using cAMP ELISA and was similar between PG-WT and PG-S665A keratinocytes (Supplemental Figure 2D). After 2 hours, PG was present in clusters along cell borders in both PG-WT and PG-S665A murine keratinocytes, whereas PG levels were reduced in PG-S665A after 6 hours (Figure 4, A and B). Comparably, DP was diminished after 6 hours of Ca^2+^-induced desmosome assembly in PG-S665A keratinocytes (Figure 4, A and C). Taken together, these data indicate that keratinocytes phospho-deficient for PG at S665 initiate desmosome assembly but fail to maintain stable DP and PG along cell-cell contact areas.

**Figure 5:**
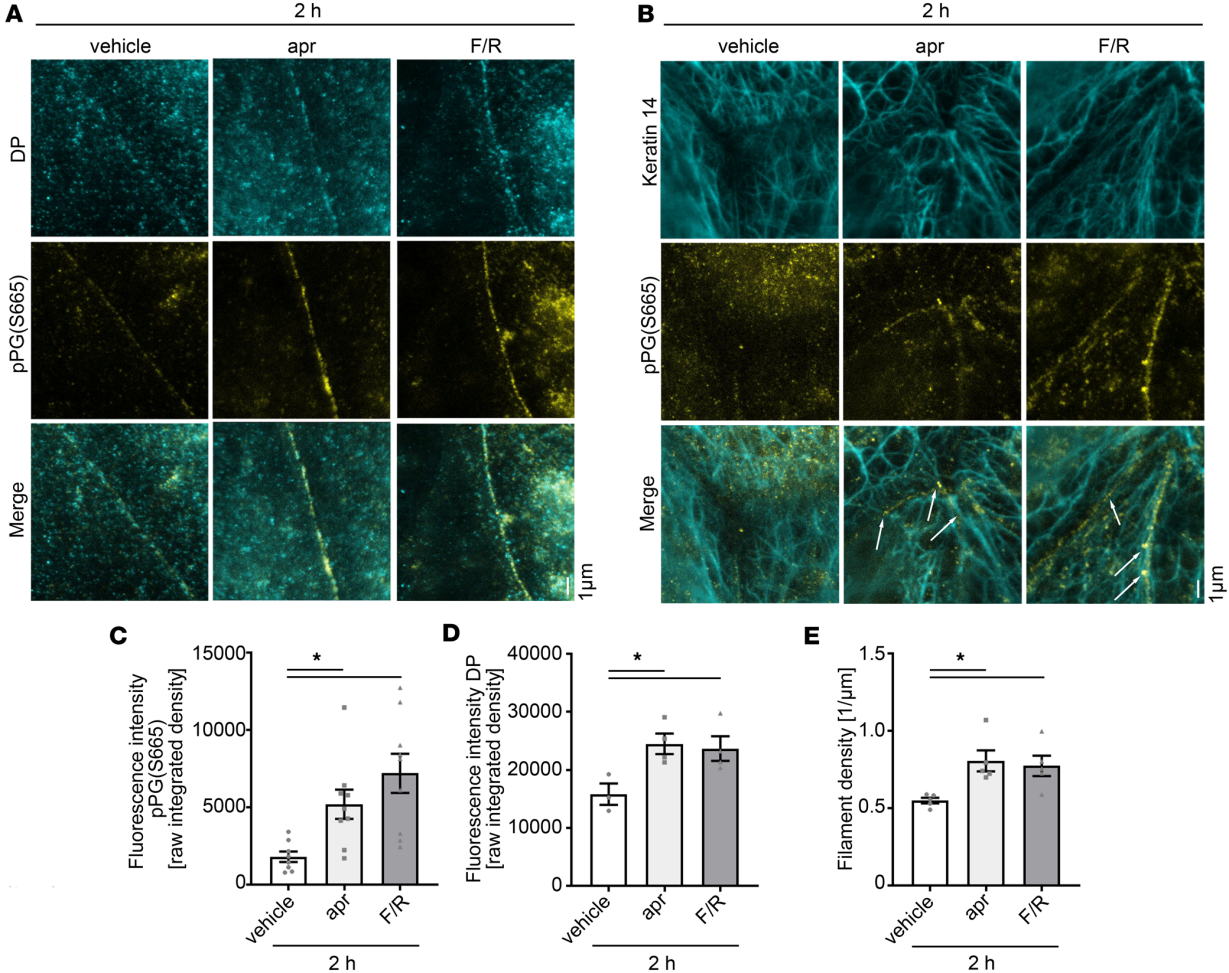
cAMP-mediated PG phosphorylation at S665 is important for proper keratin anchorage to nascent desmosomes. STED costaining of pPG(S665) and DP (**A**) or pPG(S665) and keratin 14 (**B**) in PG-WT murine keratinocyte at 2 hours after switch to high Ca^2+^ (1.2 mM) medium to allow junction assembly paralleled by incubation of either vehicle, apr, or F/R. Scale bar: 1 μm. (**A**) DP occurs as small dots along the cell border at 2 hours. DP recruitment was increased after apr and F/R treatment at 2 hours and accompanied with increased phosphorylation of PG at S665 along cell borders. Representative of *n* = 3–4. (**B**) Keratin 14 forms bundles bridging the intercellular cleft 2 hours after Ca^2+^ induction. cAMP increase by apr or F/R led to more bundles along the cell-cell contact areas, which are linked to pPG-positive areas of the cell membrane (white arrows). Representative of *n* = 5. (**C**) Quantification of pPG(S665) from **A** and **B** along cell borders showing significant increase in fluorescence intensity after apr and F/R treatment, respectively. *n* = 8–9. (**D**) Quantification of DP along cell borders showing significant increase in fluorescence intensity after apr and F/R treatment, respectively. *n* = 3–4. (**E**) Quantification of keratin filaments bridging the intercellular cleft in **B**, revealing that more filament structures inserting to the junctional areas are present in apr- or F/R-treated keratinocytes. *n* = 5. Data shown as mean ± SEM. One-way ANOVA with Dunnett’s post hoc test (**C** and **D**). Kruskal-Wallis test with Dunn’s post hoc test (**E**). *P < 0.05; apr, apremilast; DP, desmoplakin; F/R: forskolin/rolipram; PG, plakoglobin.

Given that PG phosphorylation at S665 is important for the assembly of desmosomes, we next investigated whether apremilast and F/R would promote desmosome assembly by phosphorylation of PG at S665. For this purpose, PG-WT murine keratinocytes were grown to confluency and switched to high Ca^2+^ (1.2 mM) paralleled by incubation with vehicle, apremilast, or F/R for 2 hours and 6 hours. As expected, both apremilast and F/R led to a significant increase of pPG(S665) along cell borders after 2 hours and 6 hours of assembly (Figure 5, A–C, and Supplemental Figure 3, A, B, and D). Dot-like staining of DP was present along the cell membrane after 2 hours of Ca^2+^ switch under control conditions but was found in more elongated and condensed clusters after apremilast and F/R treatment, where it colocalized to pPG(S665), indicating that formation of nascent desmosomes is accelerated upon apremilast and F/R treatment (Figure 5, A and D). In accordance, after 6 hours of Ca^2+^-induced differentiation, DP aggregates increased and linearized along cell borders under control conditions, which was even more prominent in keratinocytes treated with apremilast. A similar but not significant trend was observed after F/R treatment (Supplemental Figure 3, A and C), showing that cAMP can accelerate DP localization to the cell membrane. In addition, we observed assembly of keratin insertion during the same timeline. After 2 hours, delicate keratin bundles were observed bridging the intercellular space (Figure 5B). Interestingly, cAMP increase led to a significant increase in the number of keratin filaments inserting to the cell-cell contact area after 2 hours, which were colocalized with pPG(S665) as shown in Figure 5, B (arrows) and E. After 6 hours, keratins formed a dense mesh throughout the cell, and intercellular spaces were bridged by dense filament structures. Additionally, parallel bundles emerged comparable to those under mature conditions (Supplemental Figure 3D). Accordingly, the density of keratin fibers running parallel to the cell membrane, referred to as keratin rim, which were regarded as a sign for more mature desmosomal contacts (28), were thicker and more pronounced after cAMP increase (Supplemental Figure 3, D and E). These data indicate that cAMP-mediated PG phosphorylation at S665 is important for proper keratin anchorage to nascent desmosomes during desmosome assembly. Taken together, these data suggest that increased cAMP promotes formation of desmosomes and that phosphorylation of PG at S665 is involved in this process.

### PG phosphorylation at S665 contributes to proper adhesive function of DSG3.

**Figure 6:**
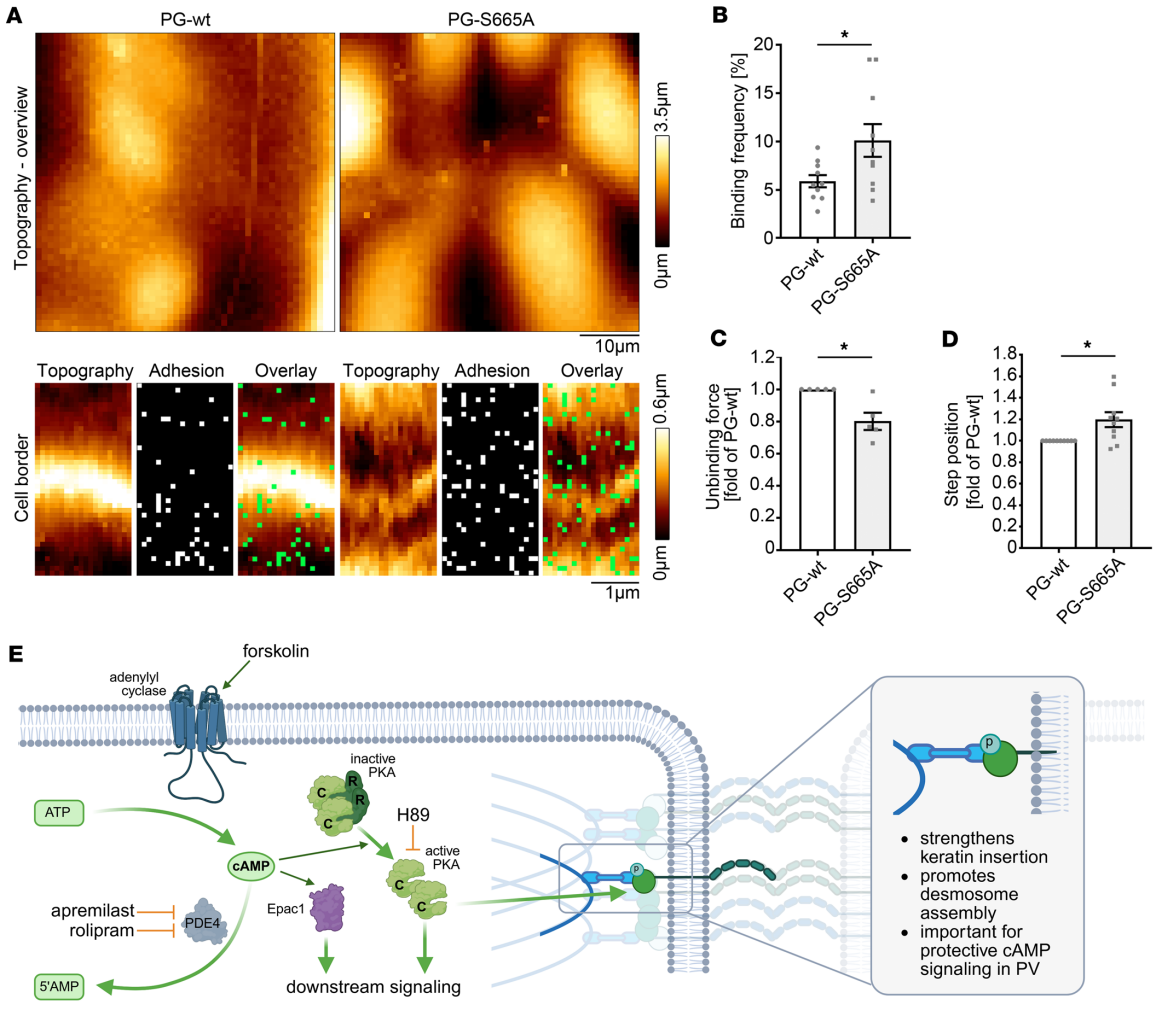
PG phosphorylation at S665 affects DSG3-dependent binding properties. (**A**) AFM experiments in PG-WT and PG-S665A keratinocytes at 48 hours Ca^2+^ differentiation. In topography images, PG-S665A keratinocytes revealed a smaller and more roundish cell shape. Cell borders revealed elevated structures with a higher slope in both cell lines. Small areas (2 × 4 μm) perpendicular to cell borders were selected for adhesion measurements. In adhesion maps, every dot represents 1 force-distance curve, and every green dot depicts 1 DSG3-dependent adhesion event. Representative of *n* = 6. Scale bars: 1 μm and 10 μm. (**B**) Quantification of binding frequency reveals a significant higher DSG3-dependent binding frequency in PG-S665A keratinocytes. (**C**) Quantification of unbinding forces showed a significantly lower binding strength of DSG3-dependent single-molecule interactions in PG-S665A keratinocytes. In every single experiment (*n*), unbinding force distribution was evaluated with peak fit using the extreme fit model. Dots represent peaks of unbinding force distribution. (**D**) Evaluation of step position revealed an increased step position in PG-S665A keratinocytes. (**B**–**D**) *n* = 6, 2 cell-borders/*n*, 800 force-distance curves/cell border. *P < 0.05, unpaired 2-tailed *t* test. (**E**) Schematic of cAMP signaling and effects of PG phosphorylation at S665. Schematic created with BioRender.

cAMP increase by apremilast prevented keratin retraction but not DSG depletion in pemphigus (15). In accordance, protein levels of DSG3 and DSG1 as well as other junctional proteins such as E-cadherin were not changed after 24 hours of apremilast incubation (Supplemental Figure 3, F–I). However, keratins also regulate the adhesive properties of desmosomal cadherins, including DSG3, as well as the mechanical properties of keratinocytes (29–31). Thus, we investigated whether the altered structure of the keratin cytoskeleton in PG-S665A keratinocytes would affect DSG3 binding characteristics and performed atomic force microscopy (AFM) experiments in PG-WT and PG-S665A after 48 hours of Ca^2+^-induced differentiation. In topography images, the different cell shape became obvious with smaller and more roundish cells in the PG phospho-deficient cell line. However, elevated cell borders indicated as white lines were present in both PG-WT and PG-S665A keratinocytes, which was probably reflected by both keratin and cortical actin filaments (Figure 6A). Next, we selected small areas of 4 × 2 μm for adhesion measurements. AFM probes were functionalized with recombinant DSG3-extracellular domain to perform DSG3-specific interaction studies. Surprisingly, DSG3 interaction probability was increased, whereas the single-molecule binding strength was reduced in PG-S665A keratinocytes (Figure 6, B and C). In addition, the step position, which represents an inverse measure for cytoskeletal anchorage (32, 33), was increased in PG-S665A cells, indicating that DSG3 molecules were less firmly attached to the keratin filament cytoskeleton (Figure 6D). To investigate whether clustering and mobility of DSG3-dependent binding events differ, we performed 5 repetitive AFM experiments along the same cell border, merged the adhesion maps, and defined clustered areas as indicated in Methods (Supplemental Figure 4A). However, neither the mobility of DSG3 nor the cluster size of DSG3-containing clusters changed, indicating they were independent of PG phosphorylation at S665 (Supplemental Figure 4, A–C).

## Discussion

Protective cAMP signaling represents an interesting therapeutic approach directly targeting keratinocyte cohesion in pemphigus (15, 22). Development of clinically approved PDE4 inhibitors such as apremilast with first successful case reports in pemphigus show the significance of this approach (16–18) and underline the importance of a concise understanding of mechanisms that lead to stabilization of keratinocyte cohesion due to cAMP increase. Previous studies in keratinocytes and cardiomyocytes reported that protective cAMP signaling leads to PKA-dependent phosphorylation of the desmosomal plaque protein PG at S665 (Figure 6E) (15, 19). Thus, we investigated the role of PG phosphorylation at S665 in desmosomal turnover and compromised desmosomal adhesion in pemphigus. We found that PG phosphorylation at S665 is crucial for the protective effects of apremilast in PV and revealed its relevance in patients with PV (Figure 6E). We observed that PG phosphorylation at S665 affects desmosome ultrastructure by enhancing keratin insertion and desmosomal assembly by augmenting the recruitment of DP and keratins to nascent desmosomal contacts (Figure 6E). Taken together, phosphorylation of PG at S665 is crucial for the protective effects of apremilast in pemphigus and for maintenance of DSG3 binding and keratin filament anchorage to desmosomes.

### Protective cAMP signaling in pemphigus involves phosphorylation of PG at S665.

Signaling pathways play a pivotal role in regulation of desmosomal adhesion in pemphigus (12). As a common principle, certain signaling pathways are dysregulated upon autoantibody binding, such as activation of p38MAPK (34), EGFR signaling (35, 36), or PLC (37, 38) and inhibition of the respective autoantibody-induced dysregulation protects keratinocytes from loss of intercellular adhesion (11, 39). In contrast, keratinocytes also respond to autoantibody binding in pemphigus with mechanisms that protect against loss of intercellular adhesion. Among them, DSG2 upregulation and cAMP signaling were recently reported (22, 40). Pemphigus autoantibodies induce a slight increase in cellular cAMP levels, which was suggested to be an insufficient rescue mechanism (22). Here, we observed that phosphorylation of PG at 665 is increased in perilesional skin from patients with pemphigus and induced by PV-IgGs after 1 hour of incubation in a human ex vivo skin model, indicating that PG phosphorylation indeed may be involved in the cAMP rescue mechanism protecting perilesional skin against blister formation. In addition, genetic database research indicates that PG-S665F (https://www.ncbi.nlm.nih.gov/clinvar/variation/1015158/) and PG-S665P (https://www.ncbi.nlm.nih.gov/clinvar/variation/811420/) variants are associated with Naxos disease whereas PG-S665 variants are known neither in pemphigus patients nor for forms of arrhythmogenic cardiomyopathy. Moreover, in cardiomyocytes, PG-S665 phosphorylation was found to be involved in cAMP-mediated strengthening of Dsg2 binding, whereas cAMP was effective in increasing cardiomyocyte adhesion independent of this mechanism (19, 41). In contrast, here we observed that PG phosphorylation at S665 is crucial for epidermal integrity, with PG-S665A mice developing epidermal micro-blistering upon mechanical stress. Pharmacological augmentation of cAMP levels is protective in pemphigus in vitro as well as ex vivo in human skin and in vivo in neonatal mice (15, 22). In accordance, we here observed that cAMP increase by apremilast had an additional effect on PG phosphorylation at S665 stimulated by PV-IgGs. Thus, we asked whether PG phosphorylation is crucial for the protective effects of apremilast in pemphigus. Indeed, in vivo experiments in neonatal mice reveal that protective effects of apremilast against PV-IgG–induced loss of intercellular adhesion are absent in PG-S665A mice, highlighting the importance of the phosphorylation at this site in protective cAMP signaling in pemphigus. This is in line with strengthening of cardiomyocyte cohesion by cAMP increase, which was paralleled by PKA-dependent PG phosphorylation at this respective site (19). However, the effect of PV-IgGs did not differ between PG-WT and PG-S665A mice, which may indicate a dose-dependent effect of PV-IgGs on PG phosphorylation. Protective cAMP signaling in pemphigus was also shown to be PKA dependent (15, 22). Along the same lines, we observed in an ex vivo human skin model that both PV-IgG–induced and apremilast-induced PG phosphorylation at S665 is, at least in part, PKA dependent. This underlines the important role for PKA in protective cAMP signaling in pemphigus. However, recent data show that further cAMP-dependent signaling pathways such as Epac1 also contribute to the protective mechanisms in pemphigus (42). Importantly, in keratinocytes, the staining pattern of keratin 14 filaments in PG phospho-deficient mice was drastically altered. Keratin 14 organization was also compromised in PV-IgG–treated PG-WT mice, which was rescued by treatment with apremilast. This is in line with previous studies where apremilast was shown to rescue keratin filament architecture in vitro in murine and human keratinocytes (15). Importantly, apremilast did not rescue keratin 14 morphology in PG-S665A mice, underlining that PG phosphorylation at S665 is crucial for the protective effects of apremilast in pemphigus.

### Organization of the keratin cytoskeleton is dependent on PG phosphorylation at S665.

Keratins are crucial for maintenance of mechanical properties of keratinocytes as well as adhesive function of desmosomes (31, 43, 44). In addition, alterations of the keratin cytoskeleton are a morphological hallmark in pemphigus and can be observed by immunostaining and in ultrastructural investigations at the single desmosome level (27). In accordance, we report here that elevation of intracellular cAMP is protective by inhibiting keratin retraction dependent on PG phosphorylation at S665. cAMP-elevating mediators increased keratin insertion in desmosomes at the ultrastructural level after 1 hour of incubation, indicating that cAMP supports keratin assembly into desmosomes, and vice versa, keratin insertion was drastically compromised at the ultrastructural level in PG-S665A keratinocytes, and cAMP-increasing mediators had no effect on the altered keratin 14 staining pattern in PG-S665A mice. Taken together, these data indicate that PG phosphorylation at S665 regulates cAMP-dependent keratin insertion. This is in line with previous studies showing that PG is required for a proper intermediate filament anchorage to desmosomes (6). Similarly, with respect to pemphigus pathogenesis, a previous study revealed that keratin retraction and PG-dependent signaling are interconnected (45).

Additionally, PG-S665A keratinocytes were previously shown to have a drastically impaired intercellular adhesion (15), similar to keratin-deficient keratinocytes (23, 46). A similarity to keratin-deficient keratinocytes also becomes evident with regard to DSG3 single-molecule binding properties. Comparable to keratin-deficient keratinocytes, PG-S665A keratinocytes revealed a higher number of DSG3-dependent binding events accompanied by lower single-molecule binding strength, showing the impact of PG phosphorylation on keratin insertion on a functional level (29, 30). Interestingly, PG-S665A keratinocytes behaved more similarly to keratin-deficient rather than to PG-deficient keratinocytes because in the latter, DSG3 single-molecule binding properties were almost unaffected (47). In addition, PG-S665A keratinocytes displayed a higher step position, indicating that anchorage of DSG3 to the cytoskeleton is impaired (32, 33).

### cAMP-dependent PG phosphorylation at S665 is involved in desmosome assembly.

Our data show that PG phosphorylation at S665 is crucial for desmosome assembly. PG-S665A keratinocytes revealed impaired recruitment of PG and DP to junctional areas. This is in line with severely altered expression of desmosomal proteins in the epidermis and keratinocytes of PG-S665A mice (15). Interestingly, PG was present in a similar amount along junctions at the early stage of desmosome assembly after 2 hours of Ca^2+^ induction, indicating that PG phosphorylation at S665 is not crucial for desmosome initiation, although various studies revealed a role for PG in initiation of desmosome assembly (48). However, PG phosphorylation at S665 affected further assembly of proper adhesive desmosomes, which is in line with previous reports showing that PG transport as well as incorporation into the cell membrane is crucial for proper desmosome assembly (49, 50). Along the same lines, PG is involved in recruitment of other desmosomal components to the cell membrane to allow desmosome formation (6, 51). Interestingly, we recently observed that apremilast does not influence assembly of desmosomes when applied 8–24 hours after Ca^2+^ induction (15). At the same time, enhancing cAMP levels with simultaneous Ca^2+^ induction led to accelerated junctional assembly, indicating that constantly elevated cAMP levels are required to speed up desmosome formation. Additionally, we observed that cAMP increase led to augmented phosphorylation of PG at S665, which was accompanied by stronger recruitment of DP to junctional areas as well as accelerated keratin insertion, indicating that the phosphorylation state of PG most likely is important for its function in desmosome assembly. We cannot rule out completely that the alterations observed in PG-S665A keratinocytes were not caused by phospho-deficiency but rather by the changes in the protein caused by altered amino acid sequence. However, since PG phosphorylation caused by cAMP-increasing mediators had contrary effects on keratin anchorage and desmosome assembly compared with the phospho-deficient mutant, these results indicate that the effects were caused by the phospho-site. Posttranslational modifications were shown to be involved in desmosome assembly, for example, phosphorylation of PKP3 and palmitoylation of PKP2 (52–54). Additionally, DP phosphorylation at S2849 by PKC also regulates DP-keratin interaction and thereby insertion of keratin filaments into desmosomes (55–57).

Although some older studies indicate that a direct interaction of PG and keratins occurs (58), the impact of PG phosphorylation on keratin filament insertion may be more indirect, either by regulation of signaling pathways such as p38MAPK (9, 30) or enhanced DP recruitment to desmosomal contacts. Here, we observed that DP recruitment to cell-cell contacts was impaired in PG-S665A keratinocytes; in addition, cAMP-increasing mediators accelerated DP clustering along cell membranes and DP colocalization to PG phosphorylated at S665. The interplay of DP and PG in the regulation of proper desmosomal adhesion was reported previously, with DP S2849 phosphorylation impairing and PG S665 phosphorylation promoting keratin insertion (13, 55, 56, 59).

Taken together, these data show the importance of PG phosphorylation at S665 for desmosome assembly as well as cAMP-dependent protective mechanisms in pemphigus. In addition, these data highlight the physiological role of cAMP signaling in the skin for a precise regulation of keratin filament organization and insertion into desmosomes.

## Methods

### Sex as a biological variable.

We used mice and ex vivo skin from both sexes. Previous studies did not provide evidence for sex-related differences at the level of desmosome biology and regulation in pemphigus. No data are available on the sex-dependent effectiveness of apremilast in pemphigus, and no or only minor sex-related differences are reported for the use of apremilast in psoriasis or Behçet’s disease (60, 61). Thus, the sex of mice used in the pemphigus neonatal mouse model was not considered as a biological variable. Donor sex and patients’ samples were not considered as a biological variable.

### Cell culture and test reagents.

Murine keratinocytes from the *Jup-S665A* mouse strain, in which Ser (S) at position 665 was substituted with alanine (A), and corresponding WT littermates were isolated using a well-established protocol (15, 23, 40). The derived cells are referred to as PG-WT and PG-S665A. For Supplemental Figure 3G, another WT murine keratinocyte cell line was used as described previously (42). To isolate the cells, the epidermis of neonatal mice was detached using Dispase II (Sigma-Aldrich) and treated with Accutase (Sigma-Aldrich) to separate the cells. Subsequently, these cells were seeded in flasks coated with collagen-I (rat tail; BD Biosciences) in complete FAD media (DMEM and Ham’s F12 Medium) (0.05 mM CaCl_2_, PAN Biotech). Immortalization occurred spontaneously after passaging the cells 10–15 times. Immortalized keratinocytes were grown in complete FAD media and switched to high Ca^2+^ (1.2 mM) at confluency for the respective time period (2 hours or 6 hours for assembly experiments; 48 hours for AFM experiments and immunostainings). Keratinocytes were maintained in a humidified atmosphere at 35°C with 5% CO_2_.

To modulate cAMP levels, we used the PDE4 inhibitor apremilast at 1 μM (Cayman Chemicals), the adenylate cyclase activator forskolin at 5 μM (Sigma-Aldrich), and the PDE4 inhibitor rolipram at 10 μM (Sigma-Aldrich). DMSO (Sigma-Aldrich) was used as a solvent for the mediators and thus applied in the same amount in the vehicle control. For Supplemental Figure 3F, apremilast was used at 100 μM.

### Purification of IgG fractions of healthy volunteers and patients with PV.

Serum samples were used with written and informed consent under approval of the local ethics committee of the University of Lübeck (AZ12-178). In the patient, pemphigus was diagnosed clinically and histopathologically. In addition, antibody profile was measured using DSG1- and DSG3-ELISA (Euroimmun). The IgG fraction was purified using protein A affinity chromatography (Life Technologies/Thermo Fisher Scientific) as described in detail before (15, 62, 63).

### Titers of PV-IgG — DSG1: 1,207 U/mL; DSG3: 3,906 U/mL.

DSG3-Fc construct, containing the full-length extracellular domain of DSG3 and a human Fc-tag, were purified for functionalization of AFM cantilevers as described before (64, 65). To do so, the construct was expressed in Chinese hamster ovary cells. Supernatants of the cells were harvested, and recombinant proteins were purified by protein A agarose affinity chromatography (Life Technologies/Thermo Fisher Scientific).

### Patient skin.

Studies involving human participants were reviewed and approved by the ethics committee of the Charite-Universitätsmedizin Berlin (ethics vote EA4/194/19). Patient biopsies were provided with written and informed consent for this study.

### Mouse strains, maintenance, and neonatal pemphigus mouse model.

This study included experiments with mice. Animal maintenance and breeding were covered by an animal breeding proposal (ethical board of Regierung von Oberbayern, ROB 55.2-2532.Vet_02-19-172). Animal experiments were approved by the ethical board of Regierung von Oberbayern (ROB 55.2-2532.Vet 02_21_205) and performed as described in detail earlier (15). For the neonatal pemphigus mouse model, WT and phospho-deficient *Jup* at S665 (PG-S665A) mice were used. The knockin mouse model for *Jup* S665A was recently established in our lab (15). Mice were maintained in a breeding facility (Max von Pettenkofer Institut, LMU Munich) in IVC System “Safe Seal” cages (Tecniplast) at 22 ± 1.5°C and a humidity of 50 ± 5% with a 12-hour light/12-hour dark cycle. Neonatal mice (2 days old) were separated from the mother and kept at 37°C. Mice were monitored using an ethical board–approved score sheet (Regierung von Oberbayern, Vet 02_21_205) and fed every 2 hours to avoid starving. Subepidermal injections were performed in the back skin of the mice using a 30G needle; 50 μL of either apremilast (1 μM) or vehicle were injected and after 2 hours, either 50 μL control-IgG (derived from healthy volunteers) or PV1-IgG were applied in a second injection. Ten hours later, mice were euthanized by decapitation, and a defined shear stress was applied to the injected area of the mice back skin (15). Skin samples were harvested and embedded for cryosectioning or fixed for electron microscopy. Tails were harvested to isolate DNA. Genotypes of the mice were determined by PCR, and the presence of the mutation was confirmed by sequencing as described elsewhere in detail (15).

To generate representative H&E and immunofluorescence stainings, sections of skin samples of mice were embedded in Tissue Tec (Leica) and cryosectioning was performed using a cryostat microtome (CryoStar NX70, Thermo Fisher Scientific). To check for occurrence of blistered skin, every 400 μm section was checked for the presence of intraepidermal blisters after staining with 1% toluidine blue solution.

### Ex vivo human skin model.

This study comprised experiments with donor skin. Experiments were performed in the context of the body donor program of the anatomy department of LMU Munich. All body donors gave their written and informed consent for the use of skin samples for research purposes. Experiments were performed as established before (66). Skin was taken from donors who were deceased no longer than 24 hours. A 30G needle was used to inject 50 μL of the respective solutions. Samples were incubated in DMEM in a humidified atmosphere with 5% CO_2_ at 37°C. For electron microscopy analysis of keratin anchorage, this solution contained either 1 μM apremilast, 5 μM or 10 μM F/R, or vehicle (DMSO 1:2,500). Samples were incubated for 1 hour and embedded in glutaraldehyde for electron microscopy analysis. For STED imaging of pPG(S665), samples were injected with either vehicle (DMSO 1:1,000), the PKA-inhibitor H89 (10 μM), apremilast (1 μM), or H89 plus apremilast and incubated for 1 hour. Subsequently, another injection of 50 μL of control-IgGs from healthy donors or PV-IgG was conducted, and skin samples were incubated another 1 hour. Samples were embedded in Tissue-Tek and cryosectioning was performed using a cryostat microtome (CryoStar NX70, Thermo Fisher Scientific).

### H&E staining.

H&E staining was performed on representative cryosections of the neonatal pemphigus mouse model according to standard procedure. Samples were mounted in DEPX (Sigma-Aldrich) and sections were imaged using a slide scanner (Mirax MIDI, Zeiss) equipped with a plan apochromat objective (×20). The digitized images were captured and viewed with the free software Slide Viewer (3DHistech; version 2.6), where blistered areas were analyzed.

### Immunostaining.

For immunostaining, representative sections of the neonatal pemphigus mouse model or skin sections from patients with pemphigus were fixed in 2% PFA for 10 minutes, permeabilized with 1% Triton X-100 for 1 hour, and treated with BSA in combination with normal goat serum (BSA/NGS) to block unspecific antibody binding. Mouse samples were incubated at 4°C with mouse monoclonal anti-CK14 (Abcam, ab7800/clone: LL002) overnight, and Cy3-coupled goat-anti-mouse secondary antibody (Dianova, 115-165-164) was applied for 1 hour at room temperature. Skin sections from patients with pemphigus were incubated with anti-pPG(S665) (custom-made; ref. 19) and anti-DP (Abclonal, A7635) antibody at 4°C overnight and subsequently with Cy3-coupled goat-anti-rabbit (Dianova, 111-225-144) and Alexa Fluor 488–coupled goat-anti-mouse secondary (Dianova, SBA-1030-30) antibodies for 1 hour at room temperature. DAPI (1 mg/mL) was added to secondary antibody incubation and probes were mounted with NPG (1% n-propyl gallate and 60% glycerin in PBS). Image acquisition was performed on a Leica SP5 confocal microscope equipped with a 63× oil objective (software version for data acquisition and analysis: 3.4.1.17822). Fluorescence intensity was measured at 10 cell borders along basal keratinocytes per mouse, and average values were used for statistical analysis. For patient samples, 10 cell borders per patient were analyzed.

### STED microscopy.

For STED experiments, murine keratinocytes were grown to confluency on #1.5 coverslips (VWR International) and switched to high Ca^2+^ medium for the time given in the respective experiments. After treatment, cells or cryosections of human skin samples were further processed with a well-established protocol (15). Briefly, fixation was accomplished using pre-cooled ethanol for 30 minutes followed by 3 minutes of ice-cold acetone on ice. BSA/NGS was used to block unspecific antibody binding. Specimens were incubated in anti-pPG (S665, custom-made; ref. 19), anti-DP (Abclonal, A7635), anti-DSG1 (Santa Cruz Biotechnology, sc-137164/clone:B-11), anti-DSG3 (ELabScience, E-AB-68435), anti-DSC3 (LS Bio, LS-B9474), and anti-PG (Progen, 690005) at 4°C in BSA/NGS overnight. Subsequently, secondary antibodies conjugated with Alexa Fluor 594 (Abcam, goat-anti-mouse, ab150120; goat-anti-rabbit, ab150084) or STAR Red (Abberior, goat-anti-mouse, 1002-500; goat-anti-rabbit,1001-500) were applied at room temperature for 1 hour. For some experiments, an anti-CK14 antibody (Abcam, ab7800/clone: LL002) was tagged with ATTO-633-NHS (ATTO Tec, AD 633-31) following the manufacturer’s protocol and was applied after secondary antibody incubation for 3 hours at room temperature to allow staining of 2 primary antibodies of the same species. Finally, cells were mounted with ProLong Diamond Antifade Mountant (Thermo Fisher Scientific). Images were taken with the Abberior Expert Line setup with STED iMSPECTOR software (version163-12585 W2040; Abberior). In accordance with previous studies, a 100×/1.4 UPlanSApo objective was used, and pixel size was set to 20 nm. To achieve the STED effect, a 775 nm pulsed laser at 20% laser power was used. Acquired images were analyzed with ImageJ (NIH) software to determine fluorescence intensity along cell borders (15, 67).

### Immunoprecipitation.

For immunoprecipitation, Protein G Dynabeads (Invitrogen, D10004D) were used to pull down Plakoglobin (Progen). The confluent monolayer of cells cultured in T75 flasks was kept on ice for 30 minutes with lysis buffer, RIPA (10 mM Na_2_HPO_4_, 150 mM NaCl, 1% (v/v) Triton X-100, 0.25% (v/v) SDS, 1% (w/v) Na-deoxycholate with adjusted pH to 7.2) in combination with cOmplete protease inhibitor cocktail (Roche Diagnostics) and PhosStop EASYpack (Roche Diagnostics). After lysing and sonication, samples were centrifuged at 4°C and 14,500*g* for 10 minutes. Supernatant was collected from each sample to measure protein concentration using a BCA standard colorimetric assay (Thermo Fischer Scientific). To perform the precleaning step, 1,000 μg of protein was mixed with lysis buffer to achieve a final volume of 1 mL and incubated with beads for 15 minutes at room temperature on rotator. The next steps were conducted based on the manufacturer’s protocol.

### Western blot.

For analysis of protein levels and immunoprecipitation, cells were either lysed in SDS lysis buffer (25 mM HEPES, 2 mMol EDTA, 25 mM NaF, and 1% sodium dodecyl sulfate, pH 7.6, protease inhibitors) or immunoprecipitation was conducted. Subsequently, electrophoresis and Western blotting were performed using standard protocols. Anti-DP (Abclonal, A7635), anti-PG (Progen, 690005), anti-DSG1 (Santa Cruz Biotechnology, sc-137164/clone: B-11), anti-DSG3 (MBL Life Sciences, D218-3/clone: AK18), anti-E-Cadherin (BD Biosciences, 610181/clone: 36), and anti–α-tubulin (Abcam, ab7291) were used at primary antibodies (overnight, 4°C) in 5% BSA and Tris-buffered saline with Tween 20. HRP-coupled secondary antibodies (1 hour, Dianova, goat-anti-mouse, 115-035-068, goat-anti-rabbit: 111-035-045) were added the next day, and blots were developed with the Western blot developer Amersham Imager 600 (Thermo Fisher Scientific).

### cAMP ELISA.

To evaluate cAMP levels in keratinocytes, a cAMP ELISA (CA-200, Sigma-Aldrich) was used. Murine keratinocytes were incubated with 1 μM apremilast for 2 hours, washed, and lysed by 0.1 M HCl. Subsequently, the cAMP ELISA was performed according to the manufacturer’s manual. Photometric measurements of color change were measured by a spectrophotometer (Tecan Plate Reader with Magellan software version V7.2).

### AFM.

Experiments were performed on a NanoWizard 3 AFM (Bruker-JPK Instruments) equipped with an inverted optical microscope (Carl Zeiss) with temperature control at 37°C. The setup allowed measurements of living keratinocytes in cell culture medium (1.2 mM Ca^2+^) and selection of scanning areas by usage of an optical image acquired with 63× objective.

For all experiments, pyramidal-shaped D-tips of Si_3_N_4_MLCT cantilevers (Bruker) with a nominal spring constant of 0.03 N/m and a tip radius of 20 nm were used and functionalized with DSG3-Fc constructs by using heterobifunctional acetal-polyethylene glycol linkers (BroadPharm) following a well-established protocol (68). Measurements were performed on living murine keratinocytes under near-physiological conditions as described elsewhere in detail (30, 65, 69). AFM was either performed in quantitative image (QI) mode for topography images or force mapping (FM) mode to characterize DSG3 binding properties. Respective settings are given in Table 1. For mobility and cluster measurements, small areas along cell borders (1 × 1 μm) were measured 5 times. Subsequently, adhesion maps were merged and color-encoded with regard to how often a binding event occurred at a certain position. A mobility coefficient was determined as described before (30, 70), and the calculation is presented in Supplemental Figure 3. Additionally, pixels that were directly attached to each other were defined as clustered, and cluster size was determined by counting the number of pixels that were attached to each other as described elsewhere in detail (70), and the calculation is presented in Supplemental Figure 3.

### Electron microscopy.

Specimens of human or murine skin were processed as described elsewhere in detail (15, 66). Briefly, skin samples of approximately 2 mm^2^ were prepared and fixed in 2.5% glutaraldehyde in PBS for 1 hour at 25°C and stored at 4°C until postfixation using 2% osmium tetroxide for 3 hours. After washing in ascending ethanol concentration and clearing with propylene oxide, samples were embedded in EPON 812 resin (SERVA Electrophoresis GmbH). Sample blocks were sliced at 60 nm thickness on an ultramicrotome (Reichert-Jung Ultracut E, Optische Werke AG) and harvested on 150 mesh copper/rhodium grids (Plano GmbH). Subsequently, sections were contrasted with uranyl acetate and lead citrate. Images were captured with a Libra 120 transmission electron microscope (Carl Zeiss NTS GmbH) equipped with an SSCCD camera system (TRS, Olympus).

### Ultrastructural quantification.

Electro-micrographs were taken along adjacent basal and suprabasal keratinocytes in human or mice epidermis. Measurement of the number of desmosomes was counted and set to a ratio to measured membrane length. Desmosome length, keratin insertion index, and keratin symmetry was measured and calculated using Fiji/ImageJ (NIH) (71) (Figure 3A). For each experiment, 30–50 desmosomes were analyzed per condition.

### Statistics.

For image processing and quantification of immunostainings and Western blot experiments, Fiji/ImageJ (NIH) was used (71). Adobe Photoshop CS5 was used to compile figures and create merged images. AFM images and data analysis of force-distance curves were done in JPK Data Processing Software (Bruker/JPK Instruments). Origin Pro (2018, 93G) was used to calculate unbinding forces, peak fitting, and step position. The free software Slide Viewer (3DHistech; version 2.6) was used to analyze the mouse epidermis for blistered areas. For all other statistics, GraphPad Prism (version 9.4.1.) was used. Shapiro-Wilk normality and Kolmogorov-Smirnov normality tests were applied to test for normal (Gaussian) distribution. For normally distributed data, a 2-tailed *t* test was used to compare 2 independent groups, whereas ANOVA (1-way or 2-way as indicated in the figures) with Tukey’s or Dunnett’s correction was applied if more than 2 groups were evaluated. As a nonparametric test, the Mann-Whitney *U* test was applied to compare 2 groups, and the Kruskal-Wallis test with Dunn’s post hoc test was applied to compare more than 2 groups. Significance was determined as *P* less than 0.05.

### Study approval.

The current study involved animal experiments, probes of patients with pemphigus, and experiments with human cadaver skin. Animal maintenance and breeding were conducted under the allowance of an animal breeding proposal (ethical board of Regierung von Oberbayern, ROB 55.2-2532.Vet_02-19-172). Animal experiments were performed under the approval of an ethical board of Regierung von Oberbayern (Vet 02_21_205). Experiments with human cadaver skin were conducted in the context of the body donor program of the anatomy department of LMU Munich. Written and informed consent for the use of skin samples for research purposes was obtained as part of the body donation program from every donor. The local ethics committee of LMU Munich gave confirmation that no further approval for the experimental protocol was required. Studies involving humans were reviewed and approved by the ethics committee of the Charite-Universitätsmedizin Berlin (ethics vote Nr.: EA4/194/19). Patient biopsies were provided with written and informed consent for this study.

### Data availability.

The datasets generated during the current study are presented in the main manuscript, supplemental material, and the Supporting Data Values file. Further information is available from the corresponding author upon reasonable request.

## Authors contributions

FV, AMS, DK, and JW were responsible for conceptualization of the study. FV, AMS, DK, JW, MH, AKEH, and MYR were responsible for the methodology. FV, AMS, DK, MH, SM, MK, MECS, PMR, TS, AKEH, MYR, DTE, LSS, KM, and KG conducted the formal analysis and investigation. FV and JW acquired funding. FV, AMS, DK, and JW supervised the study. FV prepared the original draft of the manuscript. All other authors contributed to reviewing and editing of the manuscript. All authors read and approved the final manuscript. FV and AMS share first author position. FV received the first position because she wrote the manuscript draft.

## Funding support

- Deutsche Forschungsgemeinschaft FOR 2497 (PEGASUS) to JW (TP5) and FV (TP6new).

## Supplementary Material

Supplemental data

Unedited blot and gel images

Supporting data values

## Figures and Tables

**Table 1:**
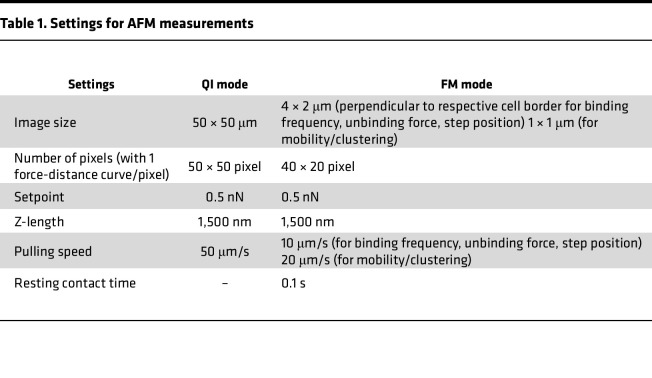
Settings for AFM measurements

## Version 1 02/09/2026

Electronic publication
