# Supplementary material for: Plakoglobin phosphorylation at serine 665 is capable of stabilizing cadherin-mediated adhesion in keratinocytes
Source: JCI Insight. 2026 Feb 9;11(3):e190359. doi: 10.1172/jci.insight.190359 (PMC12892885; doi:10.1172/jci.insight.190359)
Supplement: Supplemental data [file jciinsight-11-190359-s180.pdf]

Supplemental Material:

Fig. S1: Organization of desmosomal plaque proteins is altered in PG-S665A keratinocytes

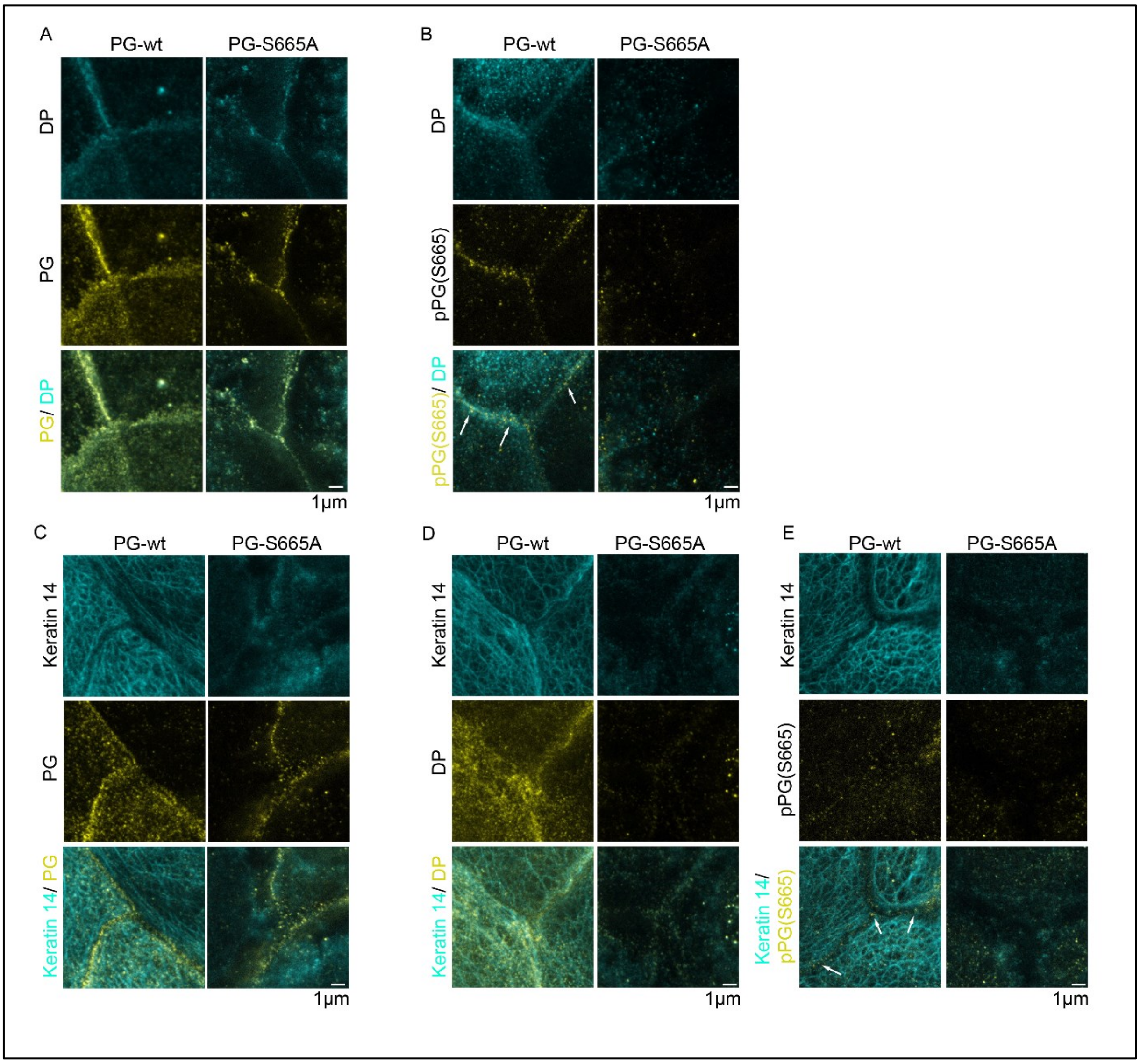

(A-E) STED images of several components of the desmosome in PG-wt and PG-S665A after 48 h of incubation in high Ca^{2+} (1.2 mM) medium. Representative of n=4. (A) Co-staining of DP and PG revealed an altered expression pattern of DP and PG, with both plaque proteins being less prominent and clustered along the cell borders in PG-S665A keratinocytes. pPG(S665) colocalized with DP (B) and membrane-inserting keratin 14 filaments (E) in PG-wt keratinocytes (white arrows). PG (C) and DP (D) colocalized to membrane-inserting keratin 14 filaments in PG-wt murine keratinocytes. (B, E) pPG(S665) is absent in PG-S665A murine keratinocytes as expected. (C-E) The keratin 14 cytoskeleton is severely disturbed in PG-S665A keratinocytes and keratin filaments inserting to cell-cell-contact areas are barely present. *PG: plakoglobin, DP: desmoplakin.*

Fig. S2: PG phosphorylation at S665 does neither change interaction to other desmosomal proteins nor reaction to apremilast

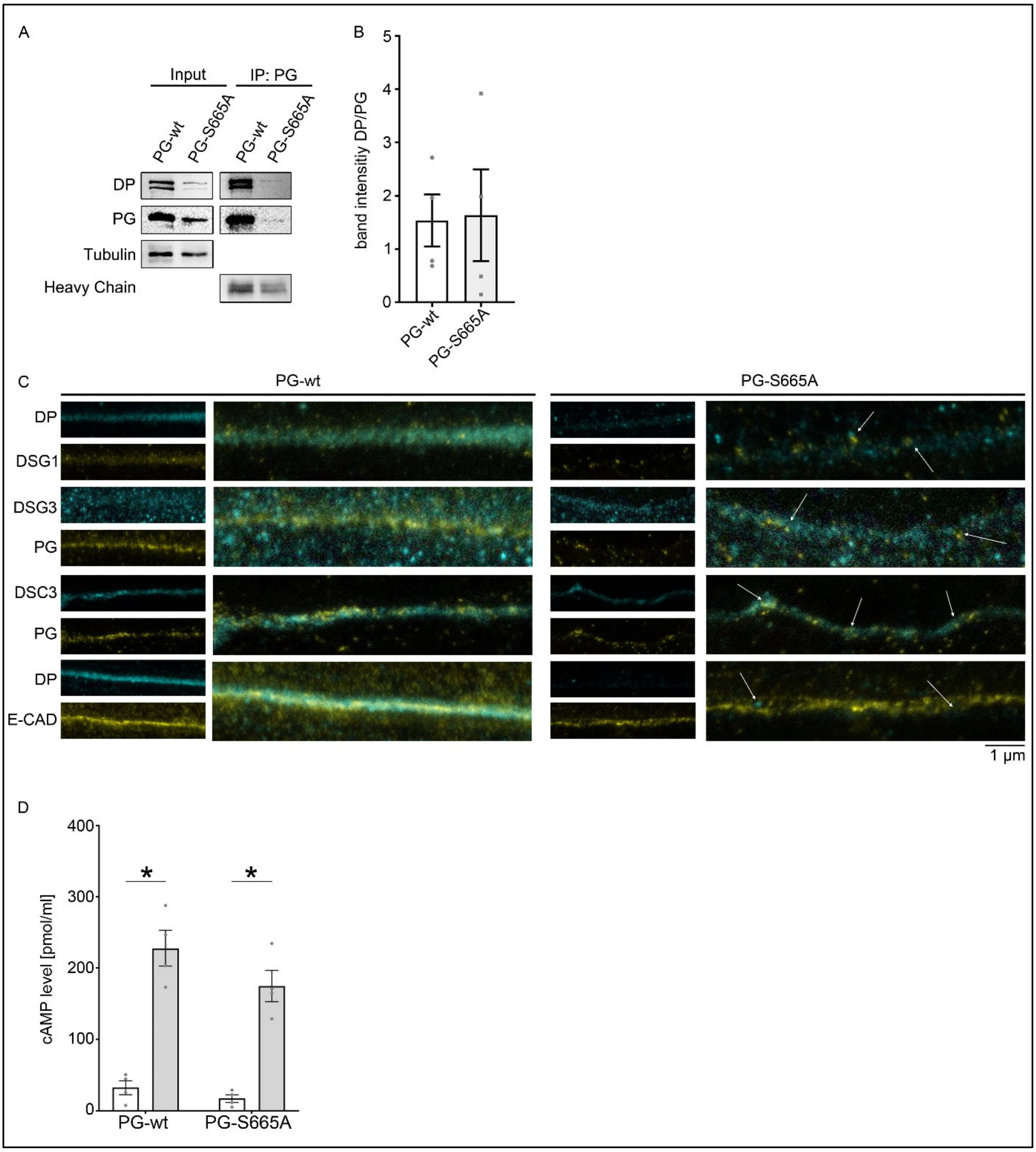

(A) Immunoprecipitation using anti-PG antibody in PG-wt and PG-S665A murine keratinocytes reveal reduced levels of PG and DP in PG-S665A keratinocytes. However, no changes in relation between PG pulldown and DP-Co-IP were observed. Representative of n=4. (B) Quantification of A showing no difference in the relative band intensity of DP/PG in the pull down. n=4. Unpaired two-tailed T-Test. *p<0.05. (C) STED imaging of DP and DSG1, DSG3 and PG, DSC3 and PG as well as E-CAD and DP showing that colocalization of desmosomal proteins are not changes between PG-wt and PG-S665A keratinocytes although total levels of PG, DP, DSG1, DSG3 and E-Cadherin (E-CAD) are reduced in PG-S665A keratinocytes. Representative of n=3. (D) cAMP ELISA revealing that baseline cAMP levels as well as apremilast-mediated cAMP increase are comparable in PG-wt and PG-S665A keratinocytes. n=4. 2-Way ANOVA, Tukey-Post-hoc test. *p<0.05. PG: plakoglobin, DP: Desmoplakin, E-CAD: E-cadherin

Fig. S3: Phosphorylation of PG at S665 impacts assembly of keratin 14 and DP to cell-cell-contact areas

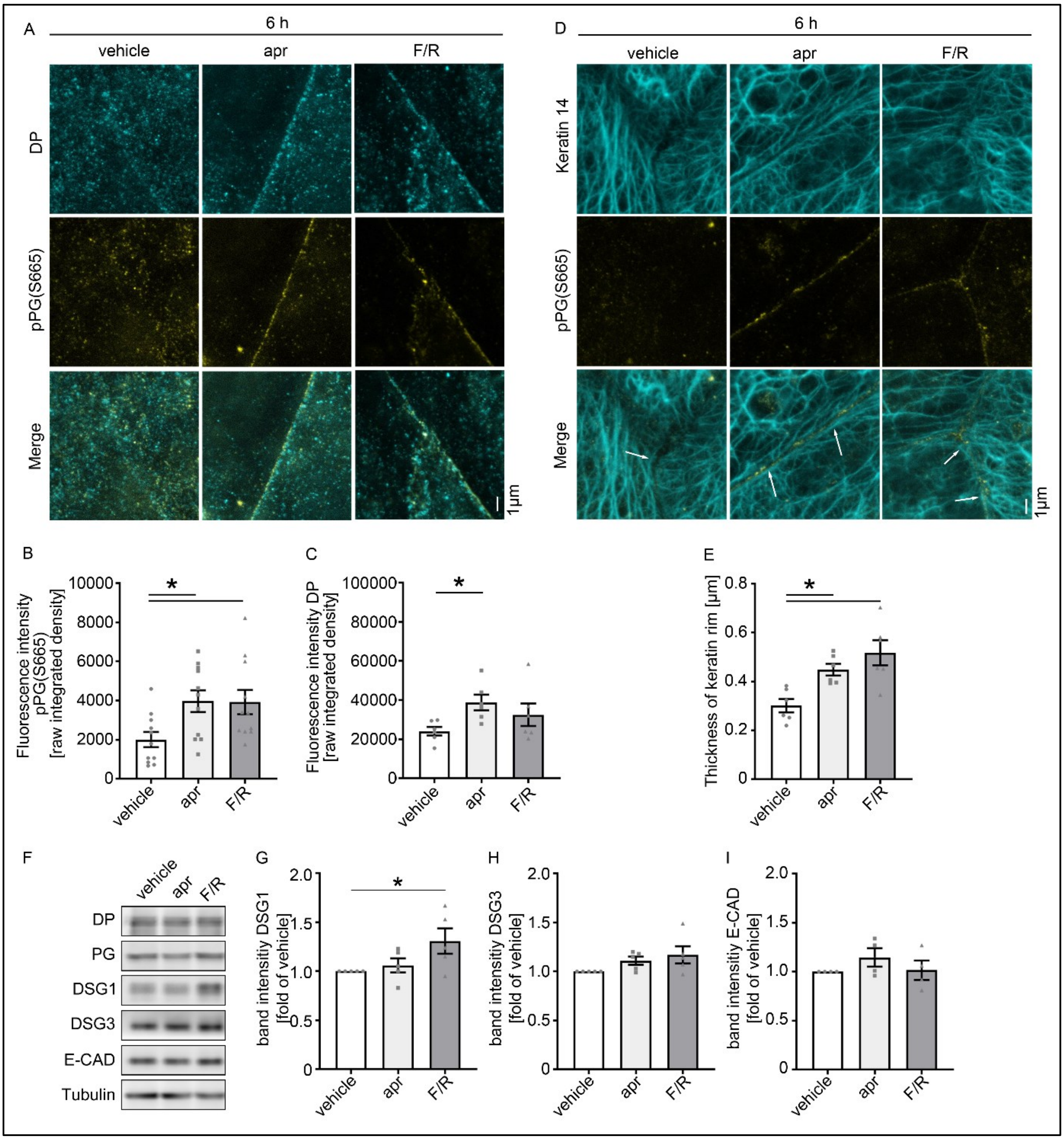

(A-E) STED experiments of desmosome assembly. Keratinocytes were switched to high Ca^{2+} medium (1.2 mM) for 6 h paralleled by a treatment of either vehicle (DMSO), apr or F/R for the same time. (A) 6 h after Ca^{2+}-induced differentiation DP clusters along the cell borders. Co-treatment with apr or F/R induced PG phosphorylation at S665 and DP staining was more linearized and organized in bigger clusters. Representative of n=5-6. (B) Quantification of pPG(S665) along cell borders showing significant increase in fluorescence intensity after apr and F/R treatment, respectively. n=11-12. (C) Quantification of DP along cell borders showing significant increase in fluorescence intensity after apr treatment. n=6. (D) Co-staining of pPG(S665) and keratin 14 in PG-wt murine keratinocyte at 6 h after switch to high Ca^{2+} (1.2 mM) medium to allow junction assembly. After 6 h of Ca^{2+} induction keratins start to form elevated structures along the cell borders with keratin bundles that run parallel to the cell membrane. This phenomenon is pronounced after cAMP increase by apr or F/R. Representative of n=5-6. (E) Quantification of (D) showing a significant increase of the keratin rim after cAMP increase by apr or F/R respectively. n=6. Bars represent mean±SEM. One-way ANOVA with Dunnett post-hoc test. *p<0.05. (F) Western blot experiments in wt murine keratinocytes after treatment with 100 µM apr or 5 µM/10 µM F/R for 24 h. Tubulin was used to measure equal load of electrophoresis gels. Apremilast did not change protein levels of DSG1, DSG3 and E-CAD. Representative of n=4-5. (G-I) Quantification of (F) showing enhanced DSG1 (G) protein levels after F/R-treatment and no differences for DSG3 (H) and E-CAD (I). Bars represent mean±SEM. One-way ANOVA with Dunnett post-hoc test. *p<0.05. *PG: Plakoglobin, DP: desmoplakin, apr: apremilast, F/R: forskolin/ rolipram.*

Fig. S4: Clustering and mobility of DSG3 is unaltered in keratinocytes phospho-deficient for PG at S665

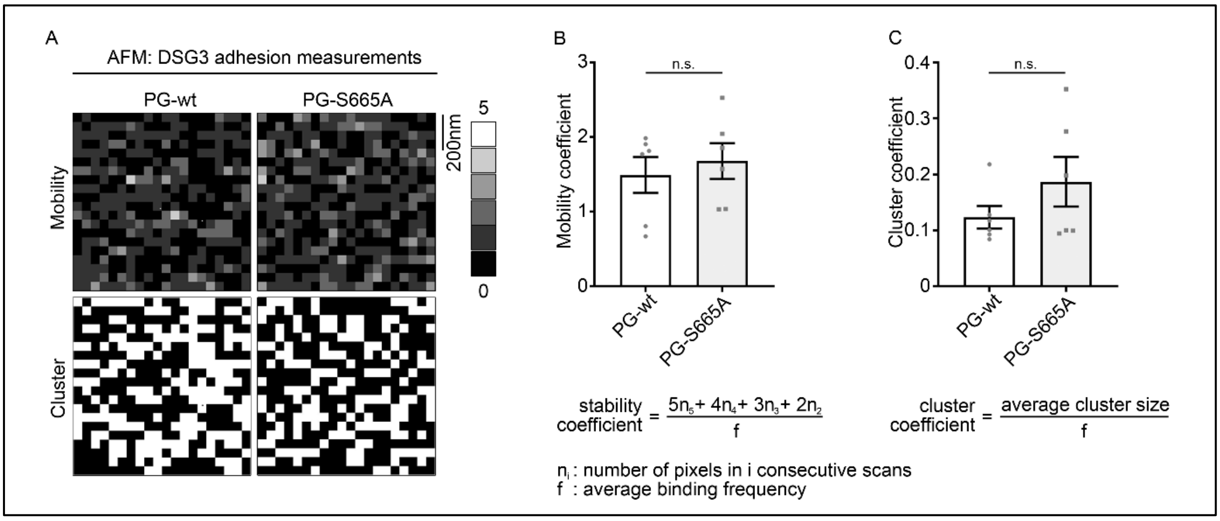

(A) AFM adhesion measurements with DSG3 coated cantilever in PG-wt and PG-S665A keratinocytes at 48 h of Ca^{2+}-induced differentiation. Small areas (1 x 1 µm) along cell borders were repetitively scanned 5 times and adhesion maps were merged. Black pixels represent areas with no DSG3-dependent binding events. Pixels of grey shades represent a certain number of scans in which a DSG3-dependent binding event occurs at this position. Attached pixels were subsequently summarized in clusters. Representative of n=3, 2 cell borders/ experiment. 400 pixels/ picture. (B) Number of stable pixels were used to calculate a mobility coefficient which was similar in PG-wt and PG-S665A keratinocytes. (C) Analysis of cluster size reveals a comparable cluster size in PG-wt and PG-S665A keratinocytes. Bars represent mean±SEM. Mann Whitney test. *p<0.05. *DSG3: Desmoglein 3*
