# Supplementary material for: Plakoglobin phosphorylation at serine 665 is capable of stabilizing cadherin-mediated adhesion in keratinocytes
Source: JCI Insight. 2026 Feb 9;11(3):e190359. doi: 10.1172/jci.insight.190359 (PMC12892885; doi:10.1172/jci.insight.190359)

Fig. S2A : anti Pg-IP, uncropped blots

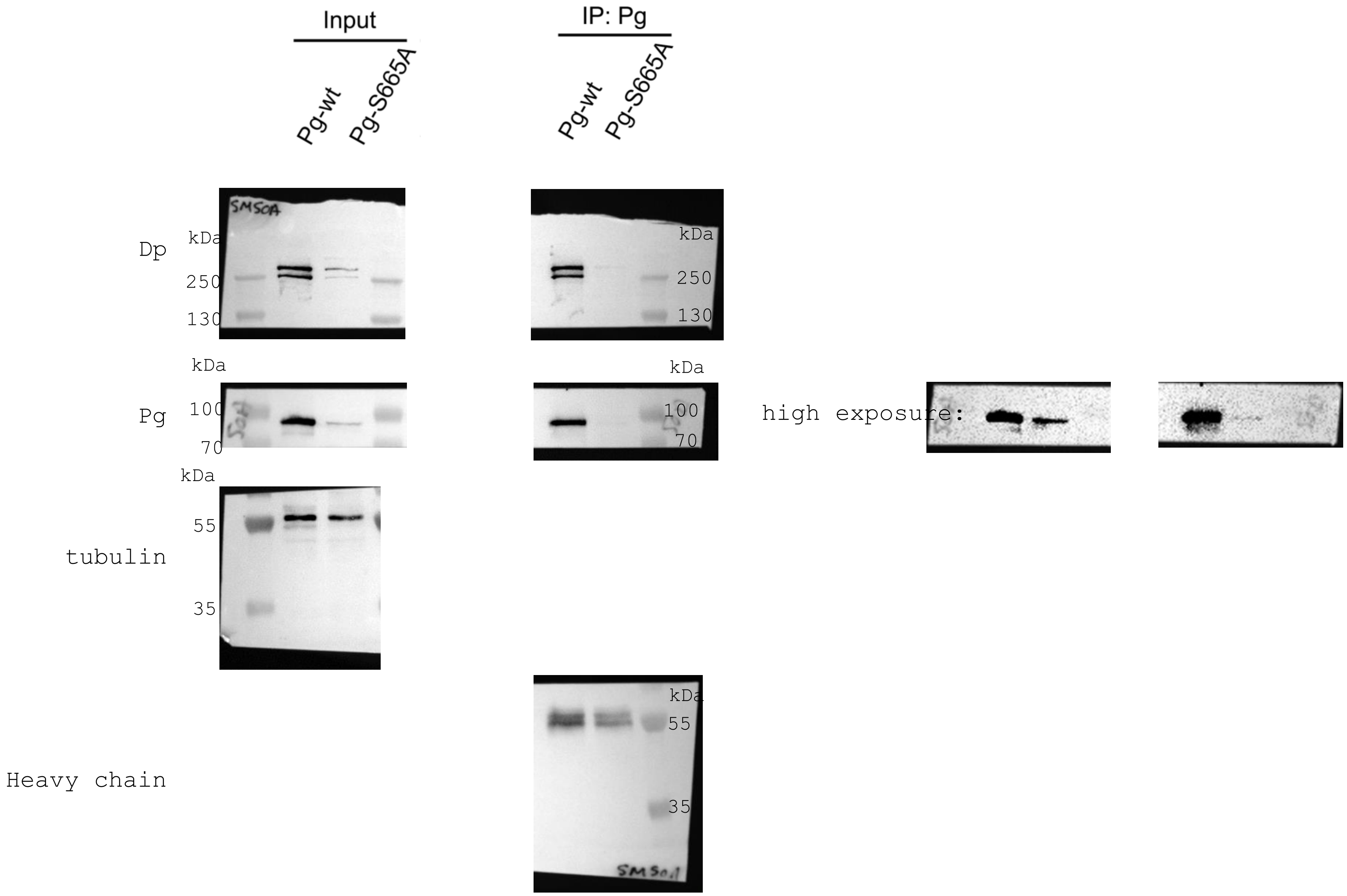

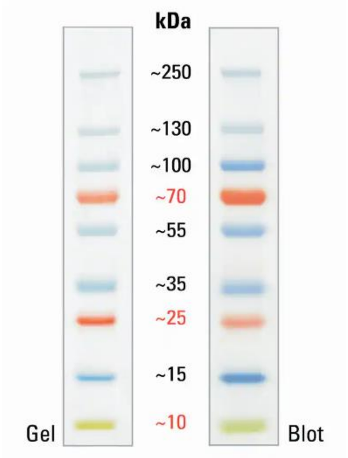

Fig.S3 F : Wb murine wt keratinocytes treated 24h with apr or F/R . uncropped blots

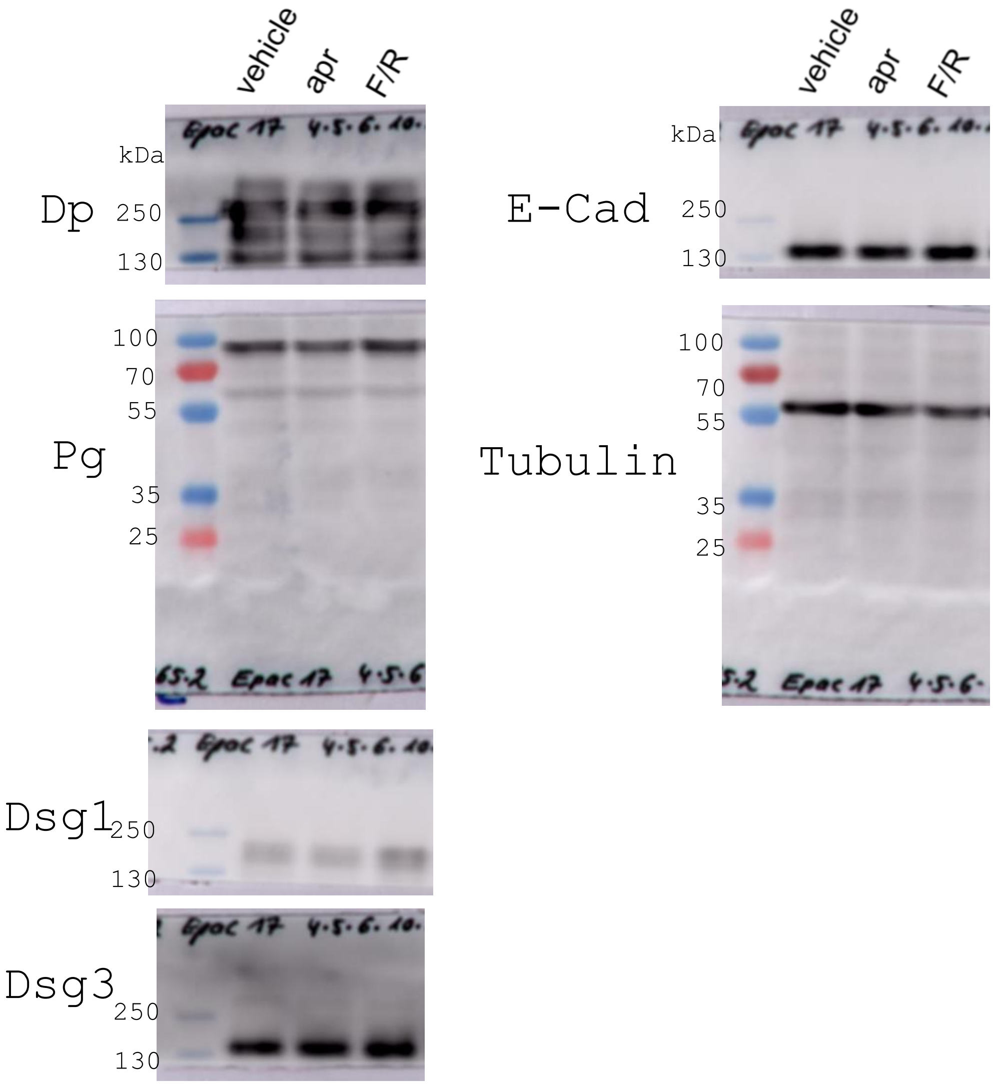

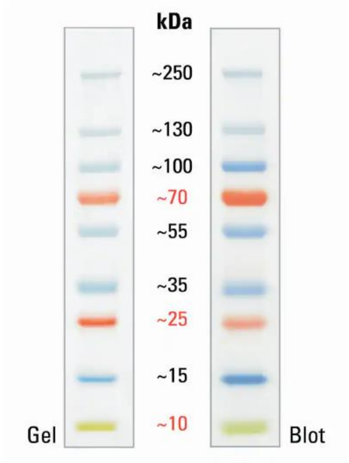

Supplement: Unedited blot and gel images [file jciinsight-11-190359-s181.pdf]
